# A novel c-Met/TRK inhibitor 1D228 efficiently inhibits tumor growth by targeting angiogenesis and tumor cell proliferation

**DOI:** 10.1038/s41419-023-06246-5

**Published:** 2023-11-09

**Authors:** Baijiao An, Wenyan Nie, Jinhui Hu, Yangyang Fan, Haoran Nie, Mengxuan Wang, Yaxuan Zhao, Han Yao, Yuanyuan Ren, Chuanchuan Zhang, Mengna Wei, Wei Li, Jiadai Liu, Chunhua Yang, Yin Zhang, Xingshu Li, Geng Tian

**Affiliations:** 1 School of Pharmacy, Binzhou Medical University, Yantai, Shandong 264003 PR China; 2 School of Biotechnology and Health Sciences, Wuyi University, Jiangmen, 529020 PR China; 3 School of Pharmaceutical Sciences, Sun Yat-Sen University, Guangzhou, 510006 PR China; 4 Shandong Technology Innovation Center of Molecular Targeting and Intelligent Diagnosis and Treatment, Yantai, Shandong 264003 PR China

**Keywords:** Drug discovery and development, Targeted therapies, Kinases

## Abstract

Multiple tumors are synergistically promoted by c-Met and TRK, and blocking their cross-signalling pathway may give better effects. In this study, we developed a tyrosine kinase inhibitor 1D228, which exhibited excellent anti-tumor activity by targeting c-Met and TRK. Models in vitro, 1D228 showed a significant better inhibition on cancer cell proliferation and migration than the positive drug Tepotinib. Models in vivo, 1D228 showed robust anti-tumor effect on gastric and liver tumor growth with 94.8% and 93.4% of the TGI, respectively, comparing 67.61% and 63.9% of Tepotinib. Importantly, compared with the combination of Larotrectinib and Tepotinib, 1D228 monotherapy in MKN45 xenograft tumor models showed stronger antitumor activity and lower toxicity. Mechanistic studies showed that 1D228 can largely inhibit the phosphorylation of TRKB and c-Met. Interestingly, both kinases, TRKs and c-Met, have been found to be co-expressed at high levels in patients with gastric cancer through IHC. Furthermore, bioinformatics analysis has revealed that both genes are abnormally co-expressed in multiple types of cancer. Cell cycle analysis found that 1D228 induced G0/G1 arrest by inhibiting cyclin D1. Additionally, vascular endothelial cells also showed a pronounced response to 1D228 due to its expression of TRKB and c-Met. 1D228 suppressed the migration and tube formation of endothelial cells, which are the key functions of tumor angiogenesis. Taken together, compound 1D228 may be a promising candidate for the next generation of c-Met and TRK inhibitors for cancer treatment, and offers a novel potential treatment strategy for cancer patients with abnormal expressions of c-Met or NTRK, or simultaneous of them.

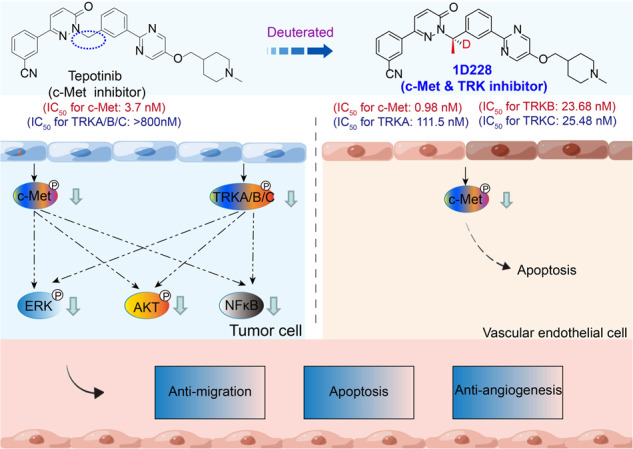

## Introduction

The c-Mesenchymal-epithelial transition factor (c-Met) is a distinct subfamily of receptor tyrosine kinases (RTKs) that is activated solely by hepatocyte growth factor (HGF) [1, 2], and plays a key role in tumor survival, growth, angiogenesis and Metastasis. c-Met overexpression and/or gene amplification occurs in a significant proportion of liver and gastric cancers [3, 4] and is associated with a high tumor stage and poor prognosis [5, 6]. Since the discovery of the HGF/c-Met pathway in the mid-1980s, c-Met has attracted significant attention as an anticancer target with several agents have been marketed including Tepotinib [7–9], Altiratinib [10], Capmatinib [11], Savolitinib [12, 13], and the ALK/c-Met inhibitor Crizotinib [14], and the initial results are encouraging. Tepotinib is a highly selective c-Met inhibitor developed by Merck, a famous German pharmaceutical company. At a concentration of 100 nM, Tepotinib is a selective inhibitor that is not able to inhibit any other kinase (besides c-Met) out of 284 kinases tested [15]. In 2021, FDA accelerated the approval of Tepotinib for marketing to treat adult patients with metastatic non-small cell lung cancer with MET exon 14 (MET ex14) jump mutation [8, 16]. Consequently, the pharmacological suppression of c-Met activity has been regarded as a burgeoning approach in cancer treatment, prompting extensive global endeavors to devise more potent and targeted inhibitors.

In cancer therapy, the tropomyosin receptor kinase (TRK) family of receptor tyrosine kinases is emerging as an important target [17]. A total of three TRK proto-oncogenes have been identified: TRKA, TRKB, and TRKC, encoded by NTRK1, NTRK2, and NTRK3, respectively [3]. Neurotrophins activate TRK receptors by binding to the extracellular region and by dimerizing, phosphorylating, and activating downstream signaling pathways, and regulate cell proliferation, differentiation and even apoptosis through the RAS/MAPKs, PI3K/AKT and PLCγ pathways [18]. Over 80 different fusion partner genes have been discovered in a wide range of adult and pediatric tumours since 1986, when an oncogenic NTRK gene fusion was discovered in colorectal cancer [19]. NTRK-fusion-positive cancers are dependent on TRK tyrosine kinase activity for growth. Several TRK inhibitors, including Entrectinib [20], Larotrectinib [21] or Repotrectinib [22, 23], have shown impressive responses in cancers with rearranged NTRK, including lung, colorectal, thyroid, neuroblastoma, gastric carcinoma, and pediatric cancers.

Various protein tyrosine kinases exhibit distinct interferences that significantly contribute to the pathogenesis of human cancer. For instance, in the context of bladder cancer, the reciprocal activation between c-Met and PDGFR, as well as Axl, actively facilitates the progression of this malignancy. Similarly, in large-cell anaplastic rhabdomyosarcoma, the HGF/c-Met pathway and c-Myc collaboratively promote the development of large cell dedifferentiated rhabdomyosarcoma. Numerous studies have demonstrated the synergistic promotion of tumor progression by c-Met and TRK kinases. For instance, in hepatoma HepG2 cells, a synergistic association exists between the HGF/c-Met and TRK signaling pathways, and the simultaneous inhibition of c-Met and TRKB exhibits a synergistic inhibitory effect on hepatoma [24]. In neuroblastoma, TRKB has the ability to up-regulate c-Met expression and augment the invasive potential of tumor cells [25]. Also, activated c-Met can enhance the activation of the TRKA signaling pathway and the biological activity of NGF [26]. The ATP analogue K252a hinders the activity of c-Met and TRK, thereby impeding the malignant characteristics of numerous tumor cells [27]. Furthermore, a separate investigation revealed that the inhibition of CDK4/6 in glioblastoma triggered the activation of the NF-κB-mediated c-Met and TrkA-B pathways. The involvement of the c-Met/TrkA-B pathway in the resistance of GBM to CDK4/6 inhibition presents a novel mechanism, suggesting that the simultaneous inhibition of c-Met/Trk should be taken into account in forthcoming clinical trials involving CDK4/6 [28]. Consequently, the inhibition of c-Met and TRK receptor kinases effectively suppresses the malignant phenotype of cancer cells.

**Fig. 1:**
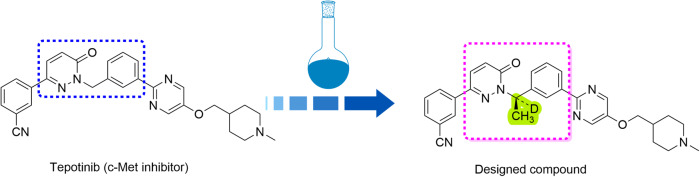
Synthesis strategy of the target compound. 1D228 was obtained by structural modification using Tepotinib as a lead.

Modification of approved anti-cancer drugs is one of the popular strategies to improve the drug efficacies. During the whole process in drug design, conformation restriction has become one of the important transformation strategies in the structural optimization of lead compounds. Proper conformation restriction can be used to control the ligand conformation and make the molecule biased to the desired conformation binding with the target proteins as too many free rotation bonds of drug molecules are not conducive for binding [29, 30]. Chirality is one of the essential properties of nature. Biological macromolecules, such as proteins, sugar, nucleic acids and enzymes, are almost chiral, and often have important physiological functions. At present, a large part of the drugs used have chirality and their pharmacological effects are achieved through strict chiral matching and molecular recognition with macromolecules in vivo [31]. The enantiomers of chiral drugs have not only significant differences in pharmacological activity but also metabolic process and toxicity in vivo [32, 33]. At present, the research of chiral drugs has become one of the main directions of international new drug research. By analyzing the molecular structure of the c-Met inhibitor Tepotinib, we believe that there is potential for optimization of rotatable chemical bonds. Therefore, we carried out the study on the introduction of chirality for restricting the conformation, and hydrogen isotope deuterium to improve its metabolic stability with Tepotinib as the lead compound (Fig. 1). Herein, we identified the excellent pharmacodynamic activity, multi-target characteristics and anti-tumor effect of the compound 1D228 as a new c-Met and TRK inhibitor candidate, which significantly suppressed tumor growth in c-Met and TRKB double expression tumors. In addition, we also found that 1D228 could inhibit both cancer cells and endothelial cells leading to improved tumor inhibition. In both gastric and hepatocellular carcinoma tumor models, 1D228 exhibited dramatic tumor suppression suggesting the potential of developing it as a c-Met and TRK inhibitor for cancer therapy.

## Results

### Synthesis of 1D228

The synthesis of 1D228 was performed according to methods in the patent with some modifications (Scheme 1). Briefly, the intermediate **3** was obtained by the coupling reaction of 3-acetylphenylboronic acid with 2-chloro-5-fluoropyrimidine in the present of palladium catalyst. The fluorine atom of intermediate **3** is replaced by N-methylpiperidine-4-methanol in the presence of sodium hydride, and then the hydrogen transfer reaction in the presence of chiral catalyst and DCOONa to afford chiral alcohol **4**. Finally, Mitsunobu reaction of chiral alcohol and pyridazinone produced the target product **1D228**.

**Fig. Scheme 1:**
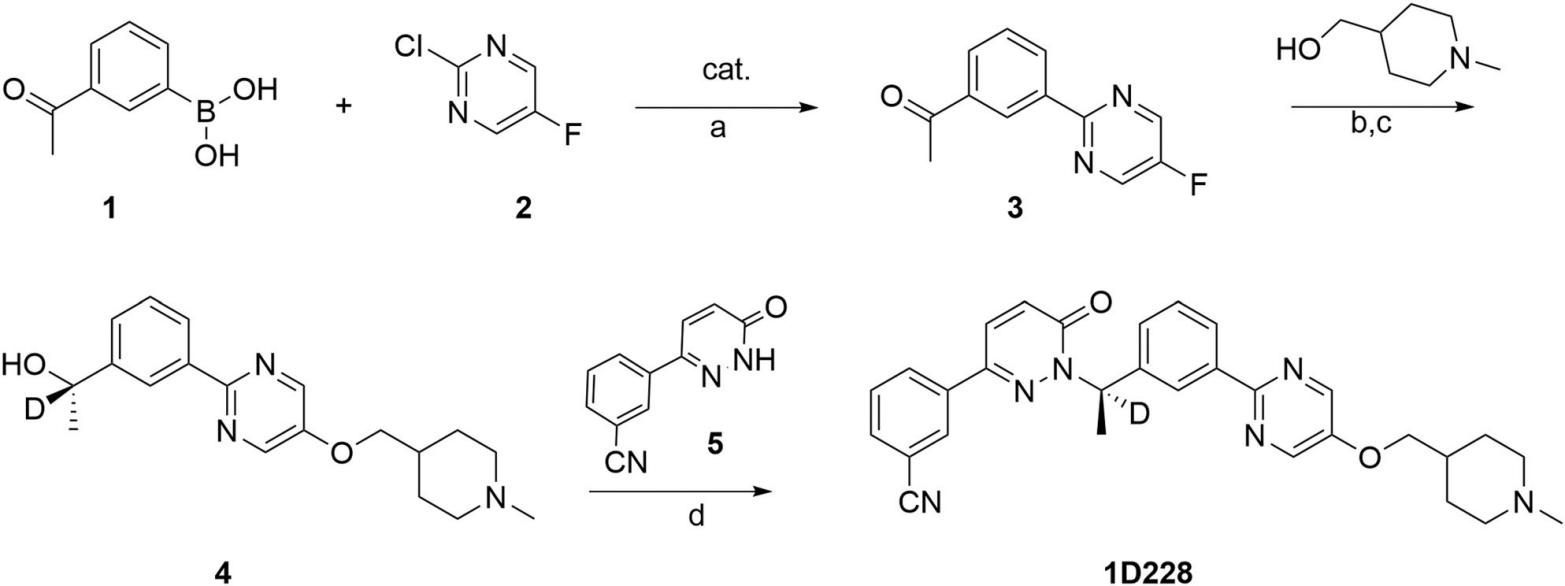
Reagents and reactive conditions. **a** bis(triphenyl phosphine)palladium chloride, sodium carbonate, H_2_0/EtOH, 83 °C, llh. **b** NaH/DMF, 1-methyl piperidine-4-methanol, 0 °C to room temperature, **c** chiral catalyst, deuterated sodium formate, rt. **d** 3-(6-oxo-l,6-dihydropyridazin-3-yl)-benzonitrile, (S)-4, triphenylphosphine, Diisopropylazodicarboxylate, 12–16 h, 0 °C to room temperature.

### Stability of compound 1D228 in liver microsome

The stability of the drug in vivo is closely related to the bioavailability and biological activity. To study the stability of 1D228, we carried out the stability assay in liver microsomes of rat, mouse, dog, monkey and human liver (Table 1). The results showed that the half-life of 1D228 was 37.19 min, 101.73 min, 63.77 min, 1653.42 min, and 2124.02 min in five models, respectively, which was better than that of Tepotinib, particularly in monkey and human liver.

**Table 1:** Metabolic Stability Evaluation of compound 1D228 and Tepotinib.

Liver microsome	T_1/2_ (min)	
	1D228	Tepotinib
SD rats	37.19	18.08
CD - 1 mice	101.73	15.14
Beagle	63.77	62.23
Macaca fascicularis	1653.42	1350.61
Human	2124.02	1829.15

### 1D228 inhibited hepatocyte cellular carcinoma and gastric cancer by targeting c-Met

**Fig. 2:**
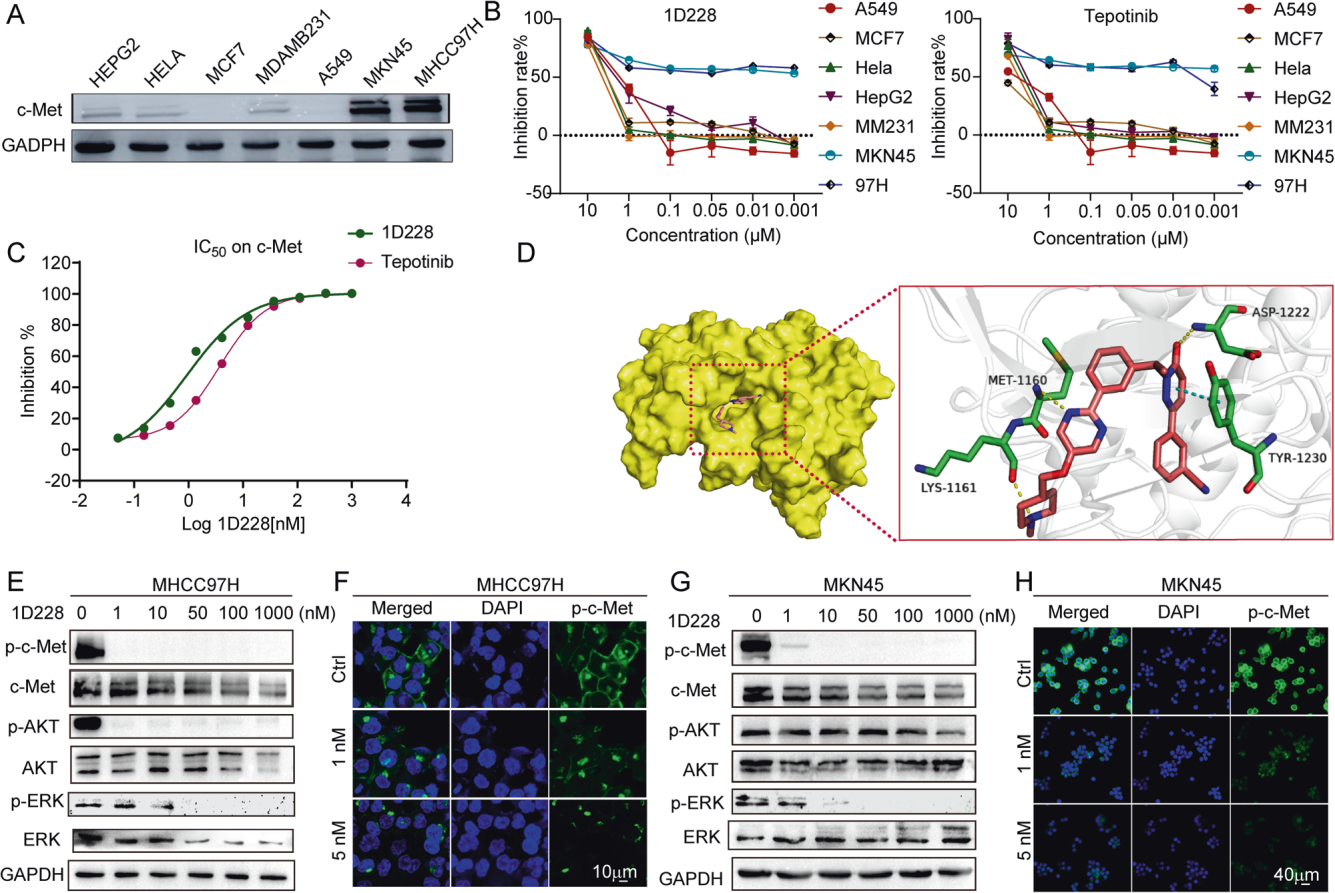
The activity of compound 1D228 on cancer cells. **A** Expression of c-Met protein in seven cancer cells. **B** Antiproliferative activity of 1D228 and Tepotinib. **C** IC_50_ of 1D228 and Tepotinib on c-Met kinase. **D** Molecular docking of 1D228 to c-Met Kinase; Western blot assay c-Met related signaling in MHCC97H (**E**) and MKN45 (**G**) cells. Immunofluorescence assay of p-c-Met protein in MHCC97H (**F**) and MKN45 (**H**) cells under 1D228 inhibition.

In order to assess the efficacy of compound 1D228 as an anti-tumor agent, we conducted immune blotting to examine the expression levels of c-Met in various cancer cell lines, including A549, MCF7, HeLa, MDAMB-231, HEPG2, MHCC97H, and MKN45. Our findings revealed that the gastric cancer cell line MKN45 and hepatocellular carcinoma cell line MHCC97H exhibited high levels of c-Met expression, whereas HepG2, Hela, and MDAMB-231 cells displayed significantly lower expression levels (Fig. 2A). These results suggest that MKN45 and MHCC97H cells possess amplification of the MET gene, leading to continuous activation of MET and reliance on oncogene addiction, which aligns with existing literature [24, 34]. This outcome suggests that MKN45 and MHCC97H cells may exhibit a more favorable response to both 1D228 and Tepotinib. Indeed, by CCK-8 assay, 1D228 selectively inhibited hepatocellular carcinoma MHCC97H (blue line) and gastric cancer cell MKN45 (cyan line) where other cancer cell lines were inhibited only at high dose (Fig. 2B). The inhibition rate goes along with the increased dose of 1D228 and Tepotinib, the IC_50_ of 1D228 were 4.3 nM or 1 nM respectively on MHCC97H or MKN45, which is lower than the reference compound Tepotinib which is 13 nM or 1.65 nM. Also, the inhibition activity test of c-Met kinase showed that 1D228 had excellent inhibition activity of c-Met kinase with 0.98 nM of the IC_50_ value comparing with the 3.7 nM of the Tepotinib (Fig. 2C). Next, in order to explore the possible binding mode of the compound and the kinase, we applied the crystal structure of c-Met as a template (PDB:4R1V) to dock with 1D228. The docking results showed that the ligand was bound to the hinge region of the kinase in a U-shape, the pyrazinone end was located inside the pocket, and the methylpiperazine ring was located outside the pocket, pointing to the solvent. Both ends are held in the pocket by forming π-π stacking with Tyr1230 and hydrogen bonding with Lys1161, respectively. The ligand occupies the ATP binding site of c-Met, competes with ATP by forming hydrogen bonds with Met 1160, and binds with Asp 1222 in the active region of c-Met to form hydrogen bonds, thus producing strong kinase inhibitory activity (Fig. 2D). In order to ascertain the inhibitory effects of 1D228 on c-Met signaling, we conducted the phosphorylation levels assay of c-Met, AKT, and ERK in MHCC97H (Fig. 2E) and MKN45 cells (Fig. 2G). Following treatment with 1D228, a significant reduction in phosphorylated c-Met was observed even at a concentration as low as 1 nM, indicating the effective inhibition of c-Met by 1D228. Additionally, the high dose of 1D228 also exhibited inhibitory effects on the phosphorylation of AKT and ERK. To further validate these findings, an immunofluorescence assay was employed to visualize the changes in phosphorylated c-Met levels in the cells. In both MHCC97H (Fig. 2F) and MKN45 (Fig. 2H) cells, treatment with 1D228 resulted in a decrease in the intensity of p-c-Met (green) compared to the vehicle treatment. Consistent with the result above, these results showed that 1D228 exhibited robust anti-cancer activity on c-Met overexpression cell lines by inhibiting c-Met kinase activity and its downstream signaling. Further investigation is required to study if this compound could be a potential new anti c-Met drug candidate.

### 1D228 showed improved anti-tumor effect compared with Tepotinib in vivo

**Fig. 3:**
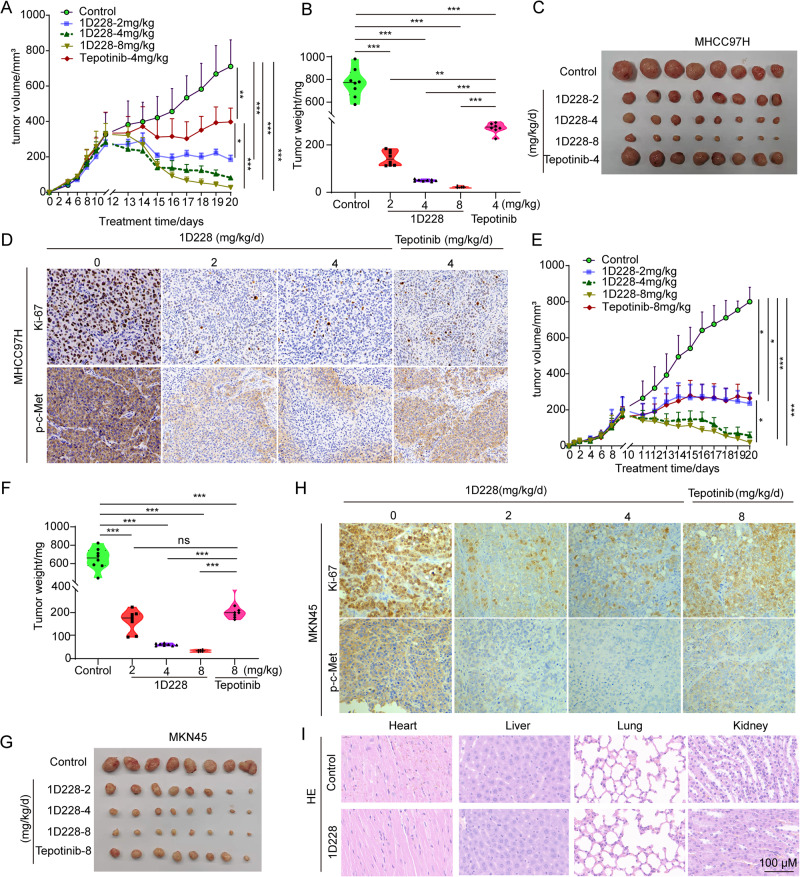
1D228 inhibited tumor growth. **A** Curve of tumor growth in MHCC97H (Data are represented as mean ± SD, *n* = 8. ***P ≤ 0.001, **P ≤ 0.01, *P ≤ 0.05); **B** Weight measurement of MHCC97H tumors (Data are represented as mean ± SD, *n* = 8. ***P ≤ 0.001, **P ≤ 0.01); **C** Images of MHCC97H tumors; **D** Ki67 staining and p-c-Met staining in MHCC97H immunohistochemistry; **E** Curve of tumor growth in MKN45 (Data are represented as mean ± SD, *n* = 8. ***P ≤ 0.001, *P ≤ 0.05); **F** Weight measurement of MKN45 tumors (Data are represented as mean ± SD, *n* = 8. ***P ≤ 0.001, n.s.: not significant); **G** Images of MKN45 tumors; **H** Ki67 staining and p-c-Met staining in MKN45 immunohistochemistry; **I** Representative images of H&E-stained sections of the heart, liver, kidney, and lung.

The data obtained from the in vitro experiment provided evidence of the strong anti-tumor cell properties of 1D228. Consequently, we proceeded to conduct in vivo studies to assess the effectiveness of 1D228 in suppressing tumor growth. In the MHCC97H xenograft tumor models, varying doses of 1D228 were administered to mice once the tumor size reached approximately 0.3 cm^3^. It is noteworthy that the administration of 1D228 resulted in a gradual regression of tumors, demonstrating a promising potential for eradication. In comparison, the positive control drug Tepotinib also exhibited a reduction in tumor size; however, the anti-tumor effect of 1D228 was significantly superior, even at the lowest dosage of 2 mg/kg/mouse (Fig. 3A). This conclusion is supported by the assessment of tumor weight and visual evidence (Fig. 3B, C). Tumor growth inhibition (TGI) was calculated through tumor weight, and 81.2%, 93.4%, 98.2% and 63.9% of that were obtained (1D288-2, 4, 8 and tepotinib), respectively. Further analysis of proliferation marker Ki67 found that 1D228 dramatically decreased Ki67 positive cells indicating the inhibition of cell growth. Again, staining results of phosphorylated c-Met also indicated a successful blockage of c-Met activity (Fig. 3D). To generalize our findings, we repeated the same settings in gastric cancer model. The MKN45 tumor cells were used to establish the tumor model. Consistent with the findings in MHCC97H tumor model, 1D228 reduced tumor size and weight, as well as showing better effect than Tepotinib (Fig. 3E–G). Notably, the low dose of 1D228 at 2 mg/kg already achieved same inhibition rate of Tepotinib at 8 mg/kg (TGI, 1D228-2mg/kg/d:74.4%; 1D228-4 mg/kg/d: 90.7%; 1D228-8 mg/kg/d: 94.8%; Tepotinib 8 mg/kg/d: 67.61%). Cell proliferation marker and p-c-Met staining was also the same trend as in MHCC97H model (Fig. 3H). Additionally, a histological examination using hematoxylin and eosin staining was conducted on major tissues (heart, liver, lung, and kidney) following various treatments and the results revealed no evidence of toxicity (Fig. 3I). In all, these results provide evidence of the remarkable efficacy of 1D228 in suppressing tumor growth without causing any discernible toxicity, surpassing the effectiveness of the current drug Tepotinib.

### 1D228 regulated gene expression and related pathway

**Fig. 4:**
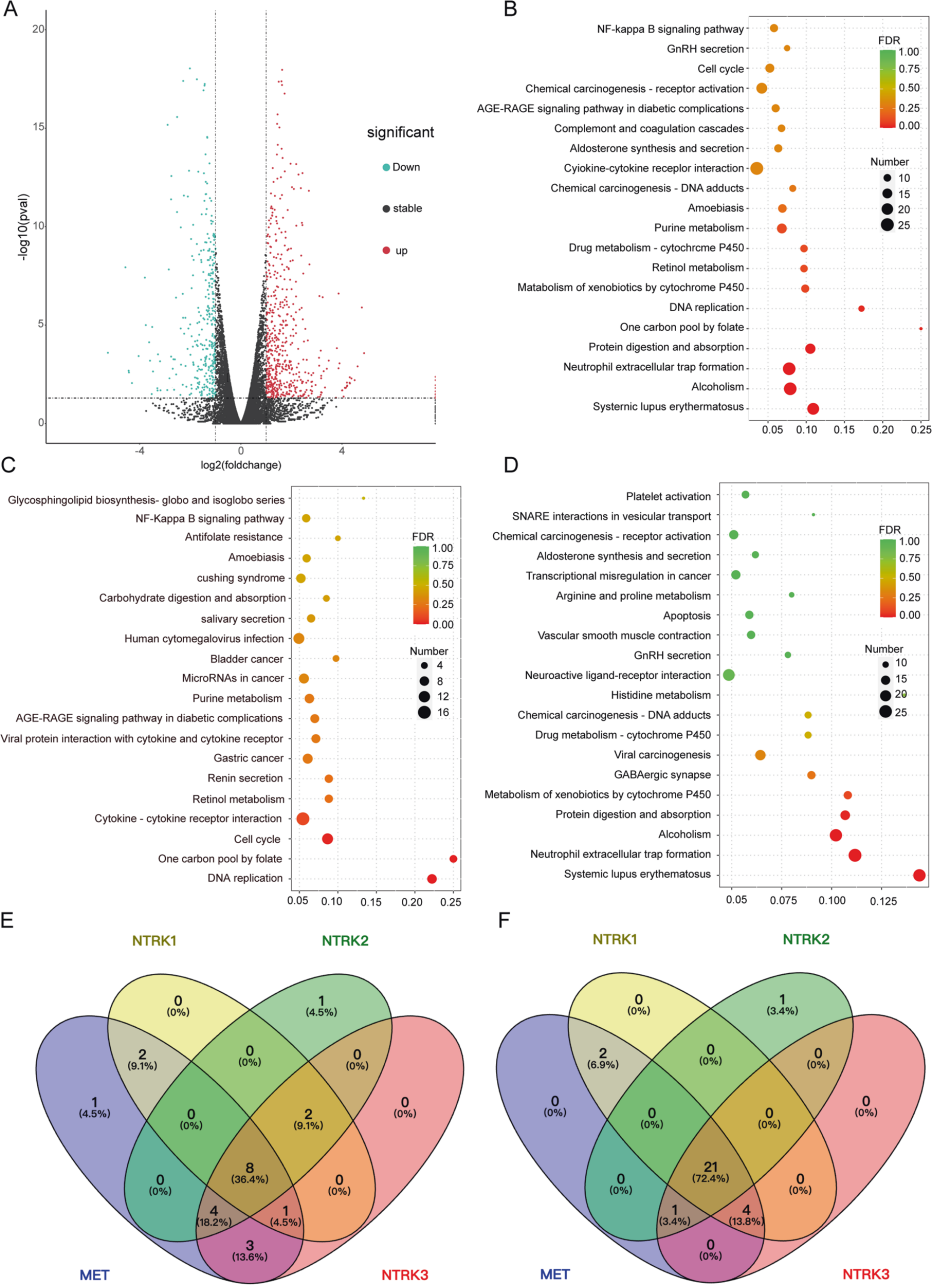
Genes and pathways regulated by 1D228. **A** Volcanogram of differentially expressed genes; **B** KEGG enriched bubble map of control vs treat differential gene; **C** Control vs treat down-regulated differential gene KEGG enrichment bubble map; **D** Control_vs_treat up-regulated gene KEGG enrichment bubble map; **E** The TCGA database reveals abnormal expression of MET, and NTRKs simultaneously in 8 cancers; **F** Significant aberrant expression of METs, and NTRKs simultaneously in 21 cancers in the TCGA merged GTEx database.

Next, in order to further elucidate the mechanism of action of this inhibitor, we conducted an analysis of gene expression in 1D228-treated MKN45 cells using RNA-seq. The results revealed that 347 genes were down-regulated and 573 genes were up-regulated following 1D228 treatment (Red dots indicate up-regulated genes in the group, blue dots indicate down-regulated genes in the group, and gray dots indicate non-significant differentially expressed genes). Multiple oncogenes were reduced in expression, including NTRK2, MYC, CEACAM18, AKR1B10, PTPRB, METTL7, etc (Fig. 4A). Moreover, 1D228 was found to induce alterations in cell cycle, apoptosis, and NF-KappaB signaling pathways as indicated by KEGG enrichment analysis (Fig. 4B–D).

NTRK2 is a member of the NTRK protein family. Numerous preclinical and clinical investigations have substantiated the role of NTRK gene fusions in driving cancer [35] and emerged as the sole tyrosine kinase exhibiting significant alterations among cancer-related genes (Fig. 4A). Moreover, it has been demonstrated that c-Met and NTRK receptors collaborate to facilitate tumor progression [24, 25]. To examine the relationship between NTRK and c-Met in major cancers, we conducted an analysis using two databases: TCGA database and TCGA combined GTEx database. The results of veen diagram (Fig. 4E, F) and upset maps (Fig. S1) showed an intersection of simultaneous and significant abnormal expression of NTRK and c-Met in multiple cancers. Among them, 8 cancer species in the TCGA database showed significant abnormal expression of both NTRK and c-Met (BLCA, GBM, KICH, KIRC, KIRP, LUAD, PRAD, and THCA, Fig. 4E,Table S1); 21 cancer species in TCGA and GTEx database showed significant aberrant expression of both of them (Fig. 4F, Table S2), namely ACC, BLCA, BRCA, CESC, COAD, GBM, KICH, KIRC, KIRP, LGG, LIHC, LUAD, LUSC, OV, PAAD, PRAD, READ, SKCM, TGCT, THCA, and UCEC. The above results suggest that 1D228 may exert anti-tumor effects as a dual target drug of c-Met and TRK, and may have clinical benefit populations.

### 1D228 targets TRK signaling and produced synergistic effect in tumor suppression

**Fig. 5:**
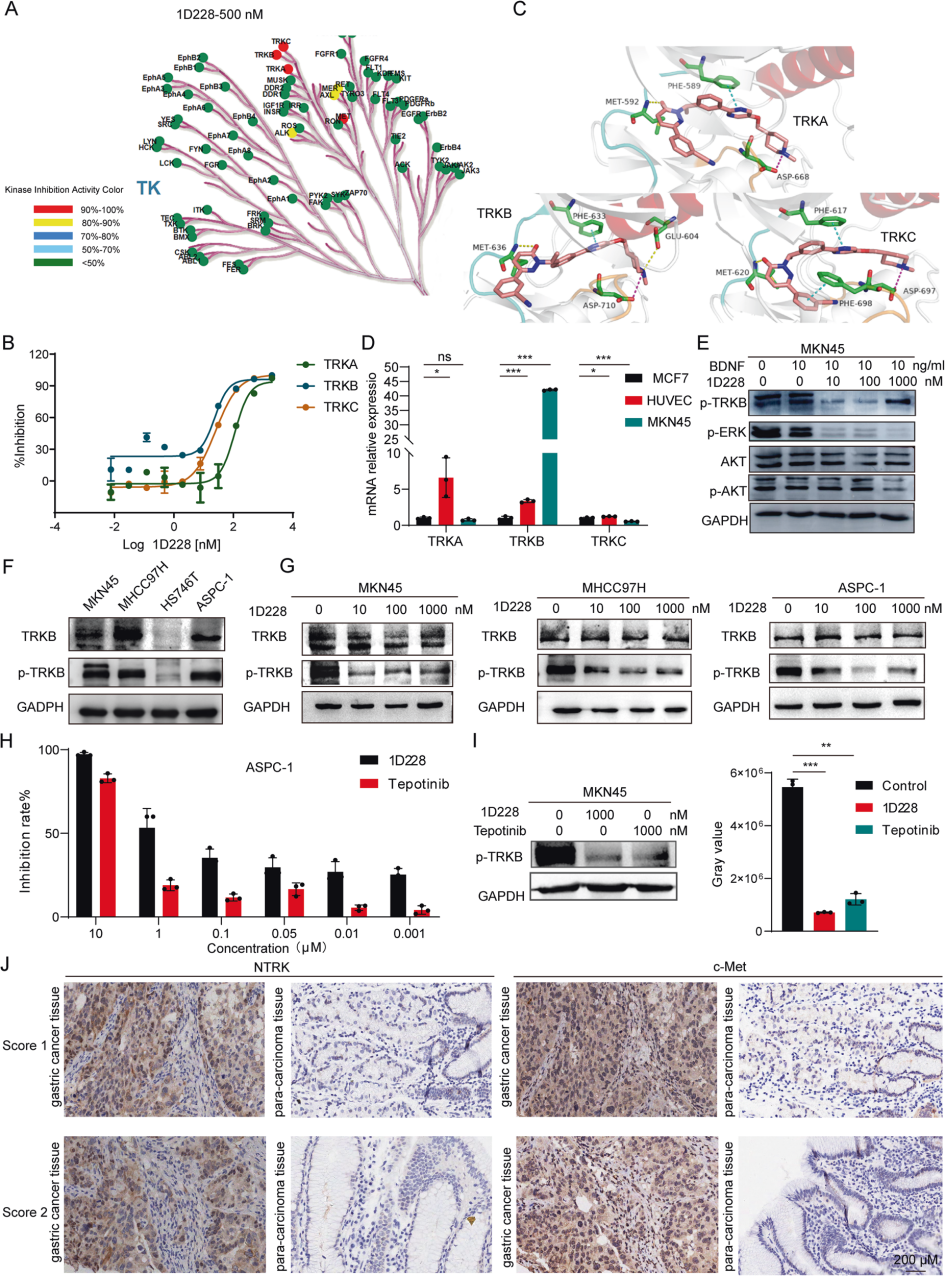
1D228 inhibits eurotrophin Receptor Kinases in both endothelial cell and tumor cells. **A** Selectivity profile of active compound 1D228 in a panel of 77 kinases; **B** Activity of compound 1D228 on TRKA, B and C kinases; **C** Molecular Docking of 1D228 with TRK Kinase; **D** TRK mRNA expression in MCF7, HUVEC and MKN45 (Data are represented as mean ± SD, *n* = 3. ***P ≤ 0.001, *P ≤ 0.05, n.s.: not significant); **E** Western blot analysis of TRKB and downstream protein phosphorylation under 1D228 treatment; **F** Expression of TRKB and p-TRKB protein in MKN45, MHCC97H, HS746T, and ASPC1 cells; **G** Effect of 1D228 on p-TRKB protein expression in MKN45, MHCC97H and ASPC1 cells; **H** Activity of 1D228 on ASPC1; **I** The effect of 1D228 on p-TRKB in MKN45 cells was tested using Tepotinib as a control (Data are represented as mean ± SD, *n* = 3. ***P ≤ 0.001, **P ≤ 0.01); **J** IHC to detect the expression of c-Met and NTRK in gastric cancer and paraneoplastic tissues.

The RNA-sep results showed that 1D228 downregulated a variety of oncogenes, so to further verify whether 1D228 also acts on other targets, we analyzed the inhibition rate of compound 1D228 against 77 TK kinases at a concentration of 500 nM using the HTRF kinase assay (Fig. 5A, Table S3). The results showed that 1D228 not only had excellent inhibitory activity on c-Met kinase, but also had certain activity on Neurotrophin Receptor Kinase (NTRK) with 111.5 nM, 23.68 nM and 25.48 nM of the IC_50_ values for TRKA, TRKB and TRKC (Fig. 5B). The TRK crystal structure was next utilized to explore possible modes of ligand docking to TRK kinase (PDB: 5JFX,4AT4,3V5Q) [36–38]. In the hinge region, the entire molecule establishes hydrogen bonds with Methionine (TRKA: MET592, TRKB: MET636, and TRKC: MET620). Additionally, the pyrimidine rings exhibit stacking interactions with gatekeeper residues (TRKA: PHE589, TRKB: PHE633, TRKC: PHE617). Within the DFG-motif, the methylpiperazine moiety forms salt bridges with aspartic acid (TRKA: ASP668, TRKB: ASP710, and TRKC: ASP697). Moreover, the addition of 1D228 induces a similar type-II binding mode in the three kinases. Specifically, 1D228 establishes hydrogen bonds with GLU604 of TRKB and exhibits π-π stacking interactions with PHE698 of TRKC. Consequently, it is plausible that the superior inhibitory activity of 1D228 against TRKB and TRKC compared to TRKA may be attributed to these molecular interactions (Fig. 5C). Afterwards, we tested the TRK mRNA expression in MKN45 and HUVEC. In comparison to MCF-7 cells, the TRKB gene was the only one highly expressed in MKN45 cells, but not in endothelial cells, where endothelial cells had both TRKA and TRKB elevated (Fig. 5D). Subsequently, we conducted an investigation into the TRKB signaling pathway in tumor cells. The utilization of western blot analysis revealed that the administration of 1D228 resulted in a reduction of phosphorylated proteins and downstream proteins within the TRKB pathway in MKN45 cells (Fig. 5E). We then assessed the expression levels of AXL, P-AXL, ALK, P-ALK, MER, and P-MER proteins in MKN45 cells following treatment with 1D228, using Tepotinib as a reference. Notably, 1D228 demonstrated significantly greater efficacy than Tepotinib in simultaneously inhibiting P-AXL, P-ALK, and P-MER expression, as depicted in Fig. S2A. Furthermore, there is evidence that MET is activated in MKN45, MHCC97H, HS746T, and ASPC1 cell lines. Consequently, we conducted separate examinations of TRKB and p-TRKB protein expression in these four cell lines. The results revealed a high expression of TRKB and p-TRKB in MKN45, MHCC97H, and ASPC1 cells (Fig. 5F). Moreover, through the use of a western blot assay, we demonstrated that 1D228 exhibited a dose-dependent inhibition of p-TRKB expression in MKN45, MHCC97H, and ASPC1 cells (Fig. 5G) and 1D228 was observed to inhibit the phosphorylation of ERK and AKT proteins in ASPC1 cells (Fig. S2B). Additionally, the efficacy of 1D228 on ASPC1 and HS746T cells was confirmed through CCK8 assay, revealing IC_50_ values of 1.33 μM (Fig. 5H) and 0.89 μM (Fig. S2C), respectively, and significantly better than Tepotinib (IC_50_: 3.78 μM and 3.6 μM). Furthermore, a comparative analysis was conducted to evaluate the therapeutic effects of 1D228 alone and in combination with Tepotinib and Larotrectinib. The results in Fig. S2D revealed that 1D228 demonstrated superior antitumor activity at an equivalent concentration (2 nM)；As reported in the liver cancer, TRKB and c-Met may have synergistic effect in promoting tumor growth [24]. To investigate this further, we examined the activity of TRKB by inhibiting c-Met using the c-Met inhibitor Tepotinib. Interestingly, the results showed that Tepotinib reduced TRKB phosphorylation, indicating that TRKB activation may be dependent on c-Met activity (Fig. 5I). Collectively, these findings provide evidence that 1D228 functions as a multi-target kinase inhibitor, effectively blocking both TRK and c-Met signaling pathways. This simultaneous inhibition may account for the superior anti-tumor efficacy observed in previous in vivo models.

Further, the co-expression of NTRK and c-Met was investigated in gastric cancer tissue microarray samples. We conducted immunohistochemistry (IHC), staining on 80 pairs of gastric cancer and adjacent normal tissues from patients, specifically targeting c-Met and NTRK using specific antibodies. Fig. 5J demonstrated a representative micrograph of the immunohistochemistry analysis. Among the 80 samples, 48 tumors tissues (60%) exhibited high expression of c-Met and 9 tumors (11.25%) exhibited high expression of Trks, comparing to the adjacent normal tissues. Additionally, co-overexpression of c-Met and TrkB was observed in 4 samples (5%) (Table S4). From above results, this study concluded that 1D228 is a double-target inhibitor of c-Met and NTRK, and may benefit patients with a variety of cancers, such as gastric, pancreatic, and liver cancer.

### Compound 1D228 induced cell cycle arrest and apoptosis

**Fig. 6:**
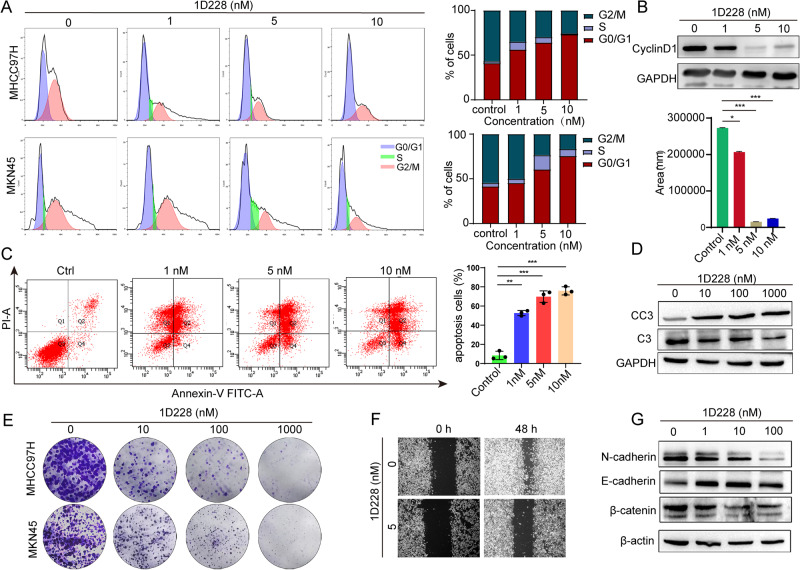
1D228 induced cell cycle arrest, apoptosis, and blocked cell migration in tumor cells. **A** Flow cytometry analysis of the cell cycle phases from MHCC97H cells and MKN45 cells under 1D228 treatment; **B** The expression of cyclin D1 by western blot (Data are represented as mean ± SD, *n* = 3. ***P ≤ 0.001, *P ≤ 0.05); **C** Apoptotic assay of MHCC97H cells using annexin V and PI staining (Data are represented as mean ± SD, *n* = 3. ***P ≤ 0.001, **P ≤ 0.01); **D** Expression of cleaved caspase-3 by western blot; **E** Colony formation assay; **F** Scratch assy of MKN45 cells under 1D228 treatment; **G** Immunoblotting of EMT markers.

KEGG enrichment analysis showed that 1D228 was involved in the cell cycle. Thus, we tested the cell cycle changes in vitro. MHCC97H and MKN45 cells were analyzed by flow cytometry. The results showed that 1D228 significantly increased G0/G1 phase in both cancer cells in a dose dependent manner (Fig. 6A). Cyclin D1 is a key molecule in the regulation of the cell cycle, and it dimerizes with CDK4/6 to regulate the transition from G1 to s phase [39]. Therefore, we tested Cyclin D1 in our cancer cell model. We found that at 5 nM and 10 nM, Cyclin D1 was substantially inhibited by 1D228 (Fig. 6B), which may be the reason for cell cycle arrest. Cycle disruption usually leads to cell death. Here we tested apoptosis using MNK45 cell line as an example. PI staining and Annexin V staining showed an accumulation of positively stained cell populations after treatment, indicating a dose-dependent apoptosis after 1D228 treatment. Statistically, 1D228 induced 53–75% apoptosis which was 10–15 times higher than in the control group (5%) (Fig. 6C). Consistent with the FACs data, western blotting results showed that there was an increase cleaved caspase-3 after 1D228 treatment, indicating more apoptotic cells (Fig. 6D). As a result, cell proliferation inhibition and increased apoptosis led to defects in colony formation of cells. Indeed, we observed fewer colonies when treated by 1D228 in MNK45 and MHCC97H cells (Fig. 6E). Not only cell proliferation suppression, 1D228 also blocked cell mobility. In the scratch assay, MNK45 cells were nearly able to close the wound by 1D228 where the control group showed a fast recovery (Fig. 6F). Similar results were seen with the invasion assay by transwell assay. In MHCC97H (Fig. S3A) and ASPC1 (Fig. S3B) cell model, 1D228 blocked 50–80% cells from migrating. The EMT pathway plays a central role in tumor migration and invasion [40]. The western blotting analysis revealed a decrease in the expression of N-cadherin and a restoration of E-cadherin upon treatment with 1D228. Additionally, the reduction in beta-catenin, a signaling molecule that governs the process of EMT, implies that 1D228 may inhibits the expression of catenin protein of WNT/beta-catenin pathway to inhibit invasion and migration (Fig. 6G). In summary, the compound 1D228 exhibited inhibitory effects on tumor cell proliferation and migration, while also inducing apoptosis.

### 1D228 impaired endothelial cell function and blocked tumor angiogenesis

**Fig. 7:**
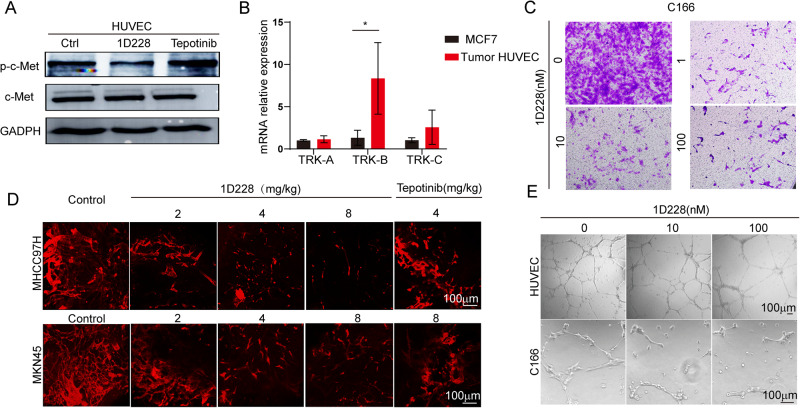
1D228 inhibited endothelial cell functions in vitro and in vivo. **A** Effect of 1D228 on p-c-Met expression in endothelial cells; **B** Relative mRNA expression of TRKs of tumor associated endothelial cells (Data are represented as mean ± SD, *n* = 3. *P ≤ 0.05); **C** Migration assay of endothelial cells; **D** Blood vessel staining of MHCC97H and MKN45 tumor tissues; **E** 1D228 inhibition affects tube formation in vitro.

It is shown that 1D228 had anti-tumor effect on tumor cells, but if this compound also targets other components in tumor tissue is unknown. To investigate this possibility, we focused on one of the typical tumor environment components, blood vessel. Angiogenesis is one of the hall marks of cancer. It plays an essential role in tumor progression in various cancers, which is regulated by many key signaling pathway, such as c-Met, AXL, or TRK, etc. [41–43]. Since in both liver and gastric tumor, we found high expression of c-Met, we would like to check if tumor associated blood vessels were also positive for c-Met, so that 1D228 might also contribute to tumor suppression via anti-angiogenesis by blocking c-Met signaling. Western blotting analysis revealed that c-Met, did express on endothelial cells. And 1D228 could inhibit p-c-Met (Fig. 7A). This data indicated that 1D228 may block endothelial cell functions. Therefore, we tested the endothelial in both in vitro and in vivo models. Tube formation is the key function of endothelial cells in building up blood vessels. We challenged both human umbilical vein endothelial cells (HUVEC) and mouse embryo endothelial cells C166 with 1D228 in the tube formation assay. The results showed that in both cells, 1D228 could inhibit tubes in a dose dependent manner (Fig. S4A). Additionally, we isolated endothelial cells from mouse MKN45 tumors for the use to investigate the expression of TRK mRNA, while tumor cell MCF7 with low TRK expression served as a control. The results revealed that murine endothelial cells exhibited higher TRK mRNA expression (Fig. 7B). Next in the migration assay, endothelial cells were nearly able to migrate (Fig. 7C). These indicated that 1D228 had significant potential in inhibiting angiogenesis. To verify this hypothesis, we administered 1D228 and Tepotinib to liver and gastric tumor bearing mice. The staining of tumor blood vessel demonstrated that both agents inhibited tumor angiogenesis, but 1D228 showed more profound suppression (Fig. 7D). Together with the in vitro data, in the presence of a certain concentration of 1D228, the vascular density of both endothelial cells decreases compared with the control (Fig. 7E), 1D228 showed a pronounced antiangiogenic property which might be the result of better anti-tumor effect.

### The in vivo xenograft model was used to test the effect of 1D228 on MKN45 tumor growth

**Fig. 8:**
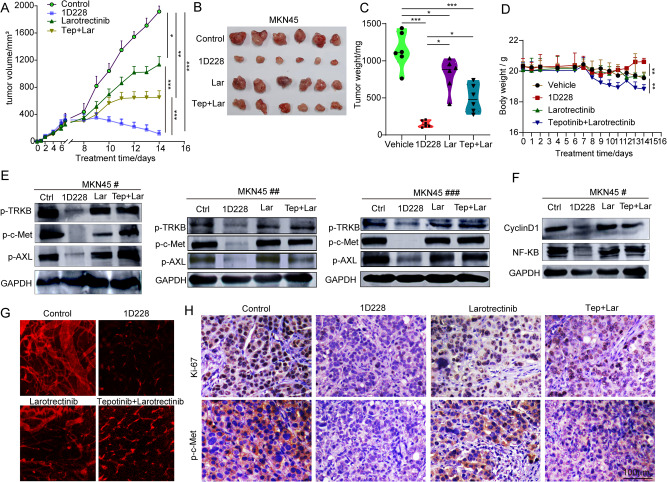
1D228 has a synergistic anti-tumor effect. **A** Tumor growth curve of MKN45 (Tep: Tepotinib, Lar: Larotrectinib; Data are represented as mean ± SD, *n* = 3. ***P ≤ 0.001, **P ≤ 0.01, *P ≤ 0.05); **B** MKN45 tumor images; **C** MKN45 tumor weight (Data are represented as mean ± SD, *n* = 6. ***P ≤ 0.001, *P ≤ 0.05); **D** Body weight of the tumor bearing mice (Data are represented as mean ± SD, *n* = 6. **P ≤ 0.01); **E** Protein level of p-TRKB, p-c-Met, and p-Axl, CyclinD1, NF-κB in MKN45 tumor tissues (*n* = 3); **F** Protein level of CyclinD1 and NF-κB in MKN45 tumor tissues; **H** Immunohistochemistry staining of ki67, p-c-Met, and p-TRK; **G** blood vessels stianing in tumor tissue.

We further compared the therapeutic efficacy of 1D228 alone with Tepotinib and Larotrectinib as a double agent in a gastric cancer nude mouse transplant tumor assay. In a gastric tumor model, 1D228 had a striking inhibitory effect on tumor growth. Under 1D228 treatment, the tumor volume shrank by nearly 90%, and the tumor began to regress after only two treatments, indicating a trend toward tumor elimination. More importantly, the combination of Tepotinib and Larotrectinib showed better tumor inhibition (56% reduction) than treatment alone (20% reduction), but 1D228 showed more significant inhibition than the combination (Fig. 8A). The tumor images and the quantification of the tumor weight also demonstrated that the pronounced anti-tumor effect of 1D228 (Fig. 8B, C). When measuring body weight in mice, the combination group showed a slight decrease in body weight, while the opposite was true for the 1D228-treated group, suggesting that the safety of 1D228 alone may be superior to the combination of Tepotinib and Larotrectinib (Fig. 8D). To validate the mechanism underlying, we examined the related protein level of p-TRKB, p-c-Met, p-AXL, Cyclin D1and NF-κB in tumor tissues of three mice. As shown in Fig. 8E, F and Fig. S4B, compared with control group, 1D228 blocked p-TRKB, p-c-Met, p-AXL, Cyclin D1 and NF-κB significantly. In comparison, larotrecinib or the combination treatment had little effect on these targets. This result, again, verified that 1D228 target TRKB, c-Met, as well as AXL signaling. Next, we evaluated the antiangiogenic effect of this treatment. Tumor angiogenesis was significantly decreased in all inhibitor-treated groups, but, in particular, 1D228 almost diminished all the blood vessels, showing a strong antiangiogenic potential (Fig. 8G). Further study on the tumor tissue showed that the expression of Ki67 and p-c-Met were all reduced by all treatments, but, again, 1D228 stands out in overwhelming inhibition on cell proliferation and c-Met inhibition (Fig. 8H). Taken together, these data demonstrated that 1D228 exerted powerful antitumor activity in vivo. By targeting c-Met and TRK-B, 1D228 could inhibit both tumor cell proliferation and tumor angiogenesis resulting in a synergistic anti-tumor effect.

## Discussion

Combing multiple drugs to treat cancer patient is a widely used strategy to achieve maximum inhibition of tumor growth and metastasis. However, this approach presents challenges such as complex pharmacokinetic properties, difficulty in dose setting, drug-drug interactions, more adverse effects, and poor patient compliance. The development of single-inhibitor with multi-target drugs is expected to improve efficacy through synergistic effects while reducing side effects. C-Met and TRK are well studied kinases in different cancers, and blocking these kinases has been demonstrated to be an effective strategy for the treatment of hepatocellular carcinoma and sarcomas with NTRK fusion. However, few studies have been investigated the dual inhibitory effect of these two targets in a same tumor model. It would be interesting to study if some tumors have both kinases under hyper activation could be super sensitive to the dual inhibition of these two targets in one shot. In this study, we found that c-Met and TRK were both activated in gastric cancer cells MKN45, indicating that blocking c-Met and TRK could be an effective strategy for treating gastric cancer. The objective of this study was to design and synthesize novel kinase inhibitors targeting c-Met and TRK as dual targets, and to investigate the effects and mechanisms of the dual-target inhibitors in c-Met and TRK high expression tumors.

Based on the structure of Tepotinib, we introduced chiral restriction conformation and hydrogen isotope deuterium to improve its metabolic stability, and synthesized a new structure of small molecule inhibitor 1D228. HTRF kinase profile analysis with a pool of 76 kinases showed that the compound 1D228 had high inhibitory bound to c-Met, TRK, AXL, ALK, and MER. Further molecular docking data indicated stable bound to c-Met and TRK. Compared with Tepotinib, 1D228 exhibited more potency on tumor cell inhibition, which may be due to the dual targeting on c-Met and TRK. The liver microsomal stability of 1D228 was significantly improved compared with Tepotinib, especially in monkey and human liver microsomes, suggesting that the compound has good metabolic stability. More surprisingly, the compounds also showed potent anti-tumor activity in vivo. In both gastric and hepatocellular carcinoma models, 1D228 regressed tumors significantly, and the tumors was largely suppressed by 1D228 comparing with the combination treatment of tepotinib and larotrectinib.

Upon 1D228 treatment, a serious of oncogenes’ expression were downregulated, and pathway analysis also indicated that the changed genes related to cell cycle, NF-κB, and DNA replication was clustered, showing that 1D228 targeted these pathways. Following experiments verified that 1D228 inhibited cell cycle in G0-G1 phase by inhibiting CyclinD1 expression, caused apoptosis by activating cleaved caspase-3. In addition to cell cycle arrest, 1D228 reversed the EMT by upregulation of E-cadherin expression and downregulation of N-cadherin, which significantly blocked the tumor cell migration. More interestingly, we found that 1D228 also targets blood vessels in both in vitro and in vivo models. Detailed investigation demonstrated that endothelial cells expressed both c-Met and TRK as well, making endothelial cells a target for 1D228. Thus, 1D228 could target both endothelial cells and tumor cells by inhibition of c-Met and TRK signaling, which produced a synergistic anti-tumor effect. This unexpected property of 1D228 makes it a promising candidate for drug development for treating cancers with both c-Met and TRK activation. In a pilot study, we have found double positive of these two kinases with high expression in clinical samples; Furthermore, bioinformatics analysis revealed co-expression of both genes in multiple types of cancer, which implies a close clinical application of 1D228 in the future. Not limited to gastric cancer, other cancers, as HCC or sarcoma patients may also benefit from this compound if double positive of c-Met and TRK are confirmed by pathological analysis.

## Conclusions

In summary, we identified 1D228 as a dual-targeting small molecule inhibitor of c-Met and NTRK, which exhibited excellent anti-tumor activity by targeting tumor cells and vascular endothelial cells simultaneously. In the nude mouse transplantation tumor model of liver cancer (MHCC97H cell lines) and gastric cancer (MKN45 cell lines), 1D228 provided splendid tumor growth inhibition rates. Considering that liver and gastric cancers are both major diseases that pose a serious threat to people’s health, and there are a large numbers of cancer patients with abnormal expressions of c-Met or NTRK, or simultaneous of them, we believe that as a multi-target kinase inhibitor, 1D228 will play an significant role in the treatment of the aforementioned cancer patients after it is approved.

## Materials and methods

### General methods

Reagents used in the synthesis were obtained commercially and used without further purification unless otherwise specified. Flash column chromatography was performed using silica gel (200−300 mesh) purchased from Qingdao Haiyang Chemical Co. Ltd. The purity of the samples was determined by high-performance liquid chromatography (HPLC), conducted on a Shimadzu LC-20AT series system with a TC-C18 column (4.6 mm × 250 mm, 5 μm). The samples were eluted with a 60:40 acetonitrile/H_2_O mixture at a flow rate of 0.5 mL/min, and was detected at a wavelength of 254 nm. The purity of all biologically evaluated compounds is greater than 95%. The ^1^H NMR and ^13^C NMR spectra were recorded using TMS as the internal standard on a Bruker BioSpin GmbH spectrometer at 400 and 100 MHz, respectively, and the coupling constants are reported in hertz. The high-resolution mass spectra were obtained using a Shimadzu LCMS-IT-TOF mass spectrometer.

#### Synthesis of (*R*)-1D228

(1) Preparation of the intermediate **3**: an aqueous solution of sodium carbonate (sodium carbonate 13.2 mmol, 1.35 g, 6.5 mL water) was added into a solution of 5-fluoro-2-chloropyrimidine (6.6 mmol, 0.87 g) in toluene (13 mL), into which were then added bis(triphenyl phosphine)palladium chloride (1.36 mmol, 0.096 g), acetyl phenyl boronic acid (6.5 mmol, 1.05 g), and ethanol (6.5 mL). Under nitrogen protection, the reaction was stirred at 83 °C for 11 h, and cooled to room temperature. 16 mL ethyl acetate and water (4 mL) were added into the reaction system to separate out the organic phase. The water phase was extracted with 5 mL ethyl acetate once. The organic phase was combined, washed with saturated brine, dried over anhydrous sodium sulfate. The solvent was removed. The crude product was added into ethyl acetate: petroleum ether (1:8), pulped, filtered and dried to get a light yellow solid 0.131 g. Yield 81%. ^1^HNMR (400 MHz, CDC1_3_), δ8.98 (t, J = 1.6 Hz, 1H), 8.70 (s, 2H), 8.59 (s,1H), 8.09 (s,1H),7.60 (s,1H), 2.71(s, 3H).

(2) Preparation of the intermediate **(*S*)-3**:

a) Preparation of a chiral catalyst: Dichloro (4-methyl isopropyl benzene) ruthenium (II) dimer (0.006 mmol, 3.7 mg), and a chiral ligand (*S, S*)-CsDPEN (0.013 mmol, 5.6 mg) were placed in a 25 mLreaction flask, into which was added water (0.6 mL) and stirred at 40°C under nitrogen protection for 4 h to prepare the catalyst, ready for use.

b) Reaction of the intermediate **3** and 1-methyl piperidine-4-methanol: At 0 °C, sodium hydride (60%, 1.5 equ, 0.376 g) was added batchwise into a solution of 1-methyl piperidine-4-methanol (1.2 equ, 6 mmol, 0.775 g) in N, N-dimethyl formamide (10 mL), and stirred for 15 min. Then a solution of the intermediate **2** (5 mmol, 1.08 g) in DMF (13 mL) was added dropwise into the above mixture while keeping the reaction temperature (0 °C) basically the same. After dropwise addition, the reaction mixture was slowly warmed to room temperature. Upon the completion of reaction monitored by TLC (about 1.5 h), the reaction solution was slowly poured into 30 mL water (with stirring), into which was added an appropriate amount of dichloromethane and stirred to separate the lower layer. The upper layer was extracted with dichloromethane. After the extraction was completed upon TLC detection, the total used amount of dichloromethane was about 40 mL. The organic phase was combined, washed with water (3*5 mL) to remove DMF as far as possible, and washed with saturated brine to obtain about 40–45 mL solution of the intermediate in dichloromethane, which was kept under nitrogen protection for use.

c) Under nitrogen protection, the above solution of the intermediate in dichloromethane was added into the prepared chiral catalyst solution, into which was then added an aqueous solution of deuterated sodium formate (1.02 g, 5 mL water, about 3 equ) under nitrogen protection. The reaction was stirred at room temperature for 10 h, until the completion of the reaction detected by TLC. 20 mL water was added into the reaction system and stirred to separate the lower layer. The water layer was extracted twice with an appropriate amount (10 mL) of dichloromethane. The organic phase was combined, washed with saturated brine, dried over anhydrous sodium sulfate, filter over a silica gel column (dichloromethane: methanol, 10:1) and eluted to get the intermediate (*S*)-4, enantiomeric excess (ee) determined by HPLC, 98% ee. Analytical conditions: AD-H column, mobile phase, isopropanol 25%, n-hexane 75% (added with 0.1% of diethylamine). The chemical purity of the intermediate (*S*)-4 was greater than 95%.

(3) Synthesis of target **(*R*)-1D228**: To a reaction flask were added 3-(6-oxo-1,6-dihydropyridazin-3-yl)-benzonitrile (2.2 mmol, 0.45 g), the intermediate (*S*)-4 (2 mmol, 0.654 g), triphenylphosphine (4 mmol, 1.05 g), and tetrahydrofuran (10 mL, with a water content not greater than 30–50 ppm), and stirred at 0°C under nitrogen protection for 10 min. Diisopropylazodicarboxylate (0.81 g, 4 mmol) was slowly added dropwise, warmed naturally to room temperature, and reacted overnight. 20 mL ethyl acetate and 10 mL water were added into the reaction system, stirred and filtered to separate out the organic layer. The water layer was extracted with 10 mL ethyl acetate. The organic phase was filtered over silica gel, and washed with ethyl acetate until no chromogenic substance was detected by TLC under ultraviolet light. Then it was eluted with ethyl acetate/petroleum ether/triethylamine (200/50/2). The eluent was collected, detected by TLC, and then concentrated to get the target product. Analytical conditions for optical purity: Daicel ADH column (0.46 × 25 cm), n-hexane (added with one thousandth of diethylamine)/ethanol = 80/20, 0.5 mL/min, λ = 254 nm, tR = 40.66 (R), tS = 64.22 (S). Analytical conditions for chemical purity:ace column, CH_3_CN:H_2_O:triethylamine = 70:30:0.1.^1^H NMR (400 MHz, CDCl_3_) δ 8.67 (t, *J* = 1.6 Hz, 1H), 8.50 (s, 2H), 8.28 (d, *J* = 7.8 Hz, 1H), 8.20 (s, 1H), 7.98 (d, *J* = 8.0 Hz, 1H), 7.69 (d, *J* = 7.8 Hz, 1H), 7.56 (m, 3H), 7.43 (t, *J* = 7.7 Hz, 1H), 7.02 (d, *J* = 9.7 Hz, 1H), 3.96 (d, *J* = 5.9 Hz, 2H), 2.92 (d, *J* = 11.4 Hz, 2H), 2.30 (s, 3H), 1.97 (m, 2H), 1.93 – 1.82 (m, 6H), 1.52 – 1.41 (m, 2H). ^13^C NMR (101 MHz, CDCl_3_) δ 159.22, 157.30, 151.70, 143.94, 141.78, 140.58, 137.89, 136.38, 132.42, 130.29, 129.82, 129.73, 129.64, 129.35, 128.75, 128.64, 127.23, 126.94, 118.57, 113.41, 73.36, 55.32, 46.44, 35.27, 28.94, 19.91. HRMS (ESI) *m/z* C_30_H_29_D_1_N_6_O_2_ [M + H]^+^, calculated:508.2566; found 508.2553. HPLC purity: 99.0%, retention time: 15.03 min. 97.1% ee. Daicel AD column (0.46 × 25 cm), n-hexane /ethanol = 80/20, 0.5 mL/min, *λ* = 254 nm, tR = 40.66 (R), tS = 64.22 (S).

#### Cell lines and culture

The human cancer cell lines (MHCC97H, HS746T and ASPC1) used in this study were purchased from the Guangzhou ginny ou biotechnology co. LTD (Guangzhou, China). The human cancer cell lines (HepG2, Hela, MCF7, A549, MKN45, MDAMB-231) used in this study were purchased from the Wuhan cell bank. Cells were identified by STR profiling, and mycoplasma contamination test were done routinely. A549, ASPC1 and MKN45 Cell lines were cultivated in 1640 containing 10% (v/v) heat-inactivated fetal bovine serum (FBS), 100 units/mL penicillin, and 100 μg/mL streptomycin. HepG2, Hela, MCF7, MDAMB-231, HS746T and MHCC97H Cell lines were cultivated in DMEM containing 10% (v/v) heat-inactivated fetal bovine serum (FBS), 100 units/mL penicillin, and 100 μg/mL streptomycin.

#### CCK8 assay

For cytotoxicity assay, the cells grown in the logarithmic phase were seeded into 96-well plates (7 × 10^3^ cells/well) for 24 h, then exposed to different concentrations (10, 5, 1, 0.1, 0.01 and 0.001 μM) of the test compounds for 72 h. After attached cells were incubated with CCK8 (Sigma, USA) for another 1–4 h. Then the absorbance at 450 nm was measured using a multifunction microplate reader (Molecular Devices, Flex Station 3), and each experiment was performed at least in triplicate. The cytotoxic effects of each compound were expressed as IC_50_ values, which represent the drug concentrations required to cause 50% tumour cell growth inhibition, and calculated with GraphPad Prism Software version 5.02 (GraphPad Inc., La Jolla, CA, USA).

#### HTRF and ADP-Glo kinase assay

HTRF kinase assay to test the activity of compound 1D228 on c-Met and NTRK kinases: c-Met, TRKA, TRKB and TRKC as well as the Zˊ-Lyte kinase Kit were purchased from Invitrogen. The experiments were performed according to the instructions of the manufacturer. Concentrations consisting of 2 or 10 levels from 0.001 to 1 mM were used for 1D228. Plate was measured on multifunction microplate reader (Molecular Devices, Flex Station 3). Curve fitting and data presentations were performed using Graph Pad Prism version 5.0. Every experiment was repeated at least 3 times.

Selectivity of 1D228 for 76 kinases based on HTRF and ADP-Glo methods: The compound was serial diluted from 10 mM stock in DMSO, dilute the test compound to 0.1 mM (200 times the specified test concentration). 2 × ATP & Substrate solution and 2 × kinase & Metal solution were prepared using assay buffer; Transfer 25 nL compound to 384 assay plate by Echo 655; After centrifugation, add 2.5 μL 2 × kinase/metal ion solution 384 assay plate, and incubate at 25 °C for 10 min. 5 μL of Kinase Detection Reagent was added to the well, and incubated for 60 min at 25 °C. 2 × XL665 & Antibody solution were prepared with detection buffer; 5 μL of Kinase Detection Reagent was added to the well, and incubated for 60 min at 25 °C; The fluorescence signals of 620 nm (Cryptate) and 665 nm (XL665) were read by microtiter plate reader; The readout value of reaction control (0.5% DMSO) was set as a 0% inhibition, and the readout value of background (10 µM Positive Control) was set as a 100% inhibition, % Inhibition = 100%-(compound-positive control)/(negative control-positive control)*100%.

#### Cell apoptosis and cell cycle assay

MHCC97H or MKN45 cells were seeded in 6-well plates (3 × 10^4^ cells/well) and incubated in the presence or absence of compound 1D228, for the cell apoptosis assay, cells were harvested and incubated with 5 μL of Annexin-V/FITC (Keygen Biotech, China) in binding buffer (10 mM HEPES, 140 mM NaCl, and 2.5 mM CaCl_2_ at pH 7.4) at room temperature for 15 min. PI solution was then added to the medium for another 10 min-incubation. Almost 10,000 events were collected for each sample and analysed by flow cytometry (Beckman Coulter, Epics XL). For the cell cycle assay, cells were fixed in ice-cold 70% ethanol overnight. After the ethanol was removed the next day, the cells were re-suspended in ice-cold PBS, treated with RNAse A (Keygen Biotech, China) at 37 °C for 30 min, and then incubated with the DNA staining solution propidium iodide (PI, Keygen Biotech, China) at 4 °C for 30 min. Approximately 10,000 events were detected by flow cytometry (Beckman Coulter, Epics XL) at 488 nm. The data regarding the number of cells in different phases of the cell cycle were analysed using EXPO32 ADC analysis software.

#### Liver microsomal stability assay

The incubation system (including 0.1 M PH 7.4 phosphate buffer, NADPH generation system, UGT incubation system, 0.5 mg/mL liver microsomal protein and appropriate concentration of drug to be tested) was incubated in a water bath at 37 °C for 200 μl. Each sample was incubated three times in parallel, and an equal volume of pre-chilled acetonitrile was added at preset reaction time points, such as 0, 5, 10, 15, 30 and 60 min. The reaction was terminated by the addition of acetonitrile. The remaining content of the prototype drug in the warm incubation solution was determined by HPLC, HPLC-MC and HPLC-MC/MC.

#### RNA isolation and RT-qPCR

mRNA expression was measured by reverse transcription quantita-tive polymerase chain reaction (RT-qPCR) on RNA isolated from cells. TRIzol reagent (Invitrogen, Carlsbad, CA, USA) was used to extract RNA from cell lines according to the manufacturer’s protocol. RNA was quantified with a NanoDrop spectrophotometer (Thermo Fisher Scientific, Waltham, MA, USA). RNA (1000 ng) was retro-transcribed to complementary DNA (cDNA) using a High-Capacity cDNA Reverse Glyceraldehyde-3-phosphate dehydrogenase (GAPDH) expression was used as an internal reference to normalize input cDNA. The threshold cycle value (CT) was determined for each measurement, and mRNA expression was calculated relative to the control using the comparative critical threshold (2 − CT) method, where CT = CTmRNA – CThousekeeping control. Triplicates were performed for each sample.

#### In vitro cell migration and invasion assay

Migration assay was performed using an 8 μm-pore Boyden chamber (24-well, #353097, Corning, NY, USA). Treated with 1, 5, and 10 nM 1D228 for 48 h. A total of 5 × 10^4^ cells were seeded in serum-free medium in the top chamber and allowed to migrate for 48 h toward the bottom chamber containing 10% fetal bovine serum medium. Cells were fixed and permeabilized with cold 70% ethanol and methanol and stained with 0.4% crystal violet. The average number of migrating cells was evaluated from at least four independent microscope fields.

#### RNA sequencing (RNA-seq) and data analysis

MKN45 cells were inoculated into large dishes at 7 × 10^4^ cells/well, and 4 nM concentration of 1D228 was added after 12 h. The compound-treated MKN45 cells and control strain cells were collected for 24 h at 37 °C in a 5% CO_2_ incubator, and three samples of each group were collected. RNA-seq and RNA-seq analysis were performed by Bioprofile Technology Company Ltd (Shanghai, China). Briefly, after RNA extraction, purification and library construction, these libraries were sequenced using Next-Generation Sequencing technology based on Illumina HiSeq sequencing platform with double-end (Paired-end, PE), and then the raw downstream data (Raw Data) were filtered. The filtered high-quality sequences (Clean Data) were compared to the human reference genome. The expression of each gene is calculated based on the results of the alignment. Based on this, the samples are further analyzed for expression differences, enrichment analysis and clustering analysis. We have uploaded RNA-seq data to the Gene Expression Omnibus (GEO) at the NCBI (National Center for Biotechnology Information).

#### Scratch assay

MKN45 or ASPC1 cells were plated in a 6-well culture dish at 5 × 10^4^ cells/dish and grown for 24 h, and the non-migrated cells were scraped off the upper surface of the membrane with a 10 ul pipette. The medium was then replaced with 10% serum 1640 medium and treated with compound 1D228 at the indicated concentrations for another 24 h. After being washed with phosphate buffer solution (PBS), the cell images were immediately detected by a Zeiss LSM 570 laser scanning confocal microscope (Carl Zeiss, Germany).

#### In vitro tube formation assay

HUVECs/C166 were seeded on 6-well plates and treated with 10 or 100 nM 1D228. After 48 h, cells were detached and transferred to growth factor reduced matrigel (Cultrex-3432-005-01) coated 96-well plates (10000 cells/well). Cells were imaged with microscope.

#### Whole mount assay

The fixed tumor sections were digested with 20 μg/ml protease K for 5 min. Methanol permeates at room temperature for 30 min; Wash with PBS at room temperature for 30 min; 4 °C wash overnight with 3% milk; Primary antibody was incubated overnight at 4 °C. Wash with 0.3%Triton-100 at 4 °C for 1.5 h; The secondary antibody was incubated at room temperature for 2 h; Incubate with 1.5% milk at room temperature for 1 h and wash overnight with 0.3% Triton-100 at 4 °C. Seal the film and shoot.

#### Extract tumor endothelial cells

Mice were dislocated and executed by cervical dislocation and then disinfected by immersion in 75% ethanol aqueous solution, then the tumor tissues were peeled, weighed, and cut by ophthalmic scissors. Collagenase solution was added and shaking was performed on a shaker for 3 min at 1700 rpm, followed by incubation in a 37 °C water bath for 5 min, alternating 10 times. The tumor tissue was digested using collagenase, and then the digested tumor tissue was centrifuged, and the supernatant after centrifugation was discarded to obtain the tissue precipitate. The precipitate was then washed in once using PBS, centrifuged after blowing the precipitate well with a pipette gun, discarded the supernatant, and repeated once. Trypsin solution was added to the collagenase-digested tumor tissue, shaken on a shaker for 10 min, and fetal bovine serum was added to terminate the digestion. The tumor cell suspension after trypsin digestion was filtered through a 30 μm filter, and the filtered tumor cell suspension was centrifuged and the supernatant was discarded; the precipitate was also resuspended and mixed with PBS, and then centrifuged and the supernatant was discarded, and the procedure was repeated once. Trypsin-digested tumor cells were resuspended by adding erythrocyte lysate, incubated at room temperature for 5 min, and the reaction was terminated by adding 5 times the volume of PBS, and the supernatant was discarded by centrifugation. The precipitate was resuspended with 1 mL of 0.5% BSA and transferred to a 1.5 mL EP tube. 10 μL of cells were resuspended with CD45 antibody and 9 times the volume of 0.5% BSA Buffer according to 10 million cells and shaken for 15 min. The cells were washed twice with 500 μL Buffer and the filtered cells were collected and centrifuged to remove the supernatant. After resuspended with CD31 and 9 times the volume of Buffer, shaken for 15 min, passed through the MACS column, Buffer washed twice, the column was taken off and 500 μLBuffer was quickly added to blow the cells into a clean 1.5 mL EP tube, centrifuged and supernatant removed, this cell is the vascular endothelial cell.

#### Colony formation assay

MKN45 cells (2 × 10^3^) and MHCC97H cells (2 × 10^3^) were seeded into the 6-well plates. After the plates were treated with 1D228 (10, 100 and 1000 nM) or DMSO (0.32% or 0.64%) for 48 h at 37 °C, then cultuerd in 10% FBS medium, the colonies were fixed with 4% paraformaldehyde and stained with 1% crystal violet solution for 20 min. The colonies were photographed and counted.

#### Western blotting

Proteins (25–50 ug/lane) were separated on a 4–12% Bis-Tris NuPAGE gel and blotted onto a PVDF membrane (FluoroTrans; VWR). Membranes were probed overnight at 4 °C with primary antibodies against p-c-Met (3077 T, Cell signaling), c-Met (8198 S, Cell signaling), p-AKT (AF0016, affinity), AKT (GR100134-1, abcam), p-ERK (AF1015, affinity), ERK (4695 T, Cell signaling), GAPDH (10494-1-AP, proteintech), p-TRKB (4603 T, Cell signaling), CyclinD1 (26939-1-AP, proteintech), cleaved caspase-3 (9661 L, Cell signaling), caspase-3 (66470-2-Ig, proteintech), N-cadherin (22018-1-ap, proteintech), E-cadherin (AF0138, Beyotime), β-actin (A5441, Sigma), AXL (4977, CST), p-MER (AF8443, affinity), P-AXL (334580, abmart), NF-KB (10745-1-AP, proteintech), p-ALK (3341 S, CST), Ki67 (27309-1-AP, proteintech). Horseradish peroxidase-conjugated secondary antibodies were from Cell Signaling. Proteins were visualized by enhanced chemiluminescence (Millipore) on ChemiDoc camera (Bio-Rad) and protein expression levels were quantified using the lmageJ software. Full anduncropped western blots are presented in Supplemental File.

#### Immunofluorescence microscopy

For the immunofluorescence microscopy, 3 × 10^5^ cells were grown in a 10 mm^3^ confocal culture dish for 24 h and then incubated in the presence or absence of compound 1D228 at theindicated concentrations for another 12 h. After being washed with phosphate buffered solution (PBS) and fixed in 4% prewarmed (37 °C) paraformaldehyde for 15 min, the cells were permeabilized with 0.5% Triton X-100 for 15 min and blocked for 30 min in 10% goatserum. Then, the cells were incubated with Rabit anti-P-MET antibody (CST, USA) at 4 °C overnight. On the next day, cells were washed with PBS three times and incubated with goat anti-Rabit IgG/Alexa-Fluor 488 antibody (Invitrogen, USA) for 1 h. After the nuclei were stained with DIPA (Sigma, USA) in the dark at room temperature for 30 min, the samples were immediately visualized on a Zeiss LSM 570 laser scanning confocal microscope (Carl Zeiss, Germany).

#### Immunohistochemistry

Immunohistochemistry (IHC) detected the expression of Ki67, P-c-Met in tumor tissue. The tumor tissue paraffin sections were deparaffinized, and endogenous peroxidase activity was blocked by incubation with 3% H_2_O_2_. The blocked sections were incubated with anti-Ki67 antibody (dilution 1:2000; proteintech 27309-1-AP dilution 1:100; Boster, A00254), anti-c-Met antibody (dilution 1:200; CST 3077) at 4 °C overnight and then sequentially incubated with a biotin-labeled secondary antibody. The sections were then stained with 3,3′-diaminobenzidine. Finally, the sections were counterstained using hematoxylin and fixed. For each section, three fields of view were randomly selected and photographed under ×200 magnification.

#### Bioinformatic analysis

To determine which cancers specifically express the MET and NTRK families (NTRK1; NTRK2; NTRK3), we downloaded RNA sequencing expression profiles for 32 cancers from The Cancer Genome Atlas (TCGA) (https://www.cancer.gov/ccg/research/genome-sequencing/tcga) and The Genotype-Tissue Expression (GTEx) dataset (https://gtexportal.org/home/). We then used *VENNY2.1* [44] (https://bioinfogp.cnb.csic.es/tools/venny/), an online analysis site, to visualize the intersection of cancers with significantly (*p* < 0.05) abnormal expression of MET and NTRK family genes.

#### Detection of c-Met and TRK protein expression in gastric cancer tissue microarrays using IHC

Human gastric cancer tissue microarrays (containing 80 cases of gastric cancer tissues and corresponding paracancerous tissues, HStmA160CS01) were purchased from Shanghai Xinchao Biological Co., Ltd. and serially sectioned tissue microarrays were selected and incubated and DAB stained with c-Met (dilution 1:50; CST 8198 S) and TRK (dilution 1:50; SC 8058) antibodies, respectively, after dewaxing, antigen repair and sealing steps.

#### In vivo animal experiments

Male BALB/c nude mice (5-week old, 18–20 g) were purchased from Beijing Vital River Laboratory Animal Technology (Beijing Vital River LaboratoryAnimal Technology Co., Ltd., Beijing,China) and allowed to acclimate for 1 week, maintained at constant room temperature and fed a standard rodent chow and water. 1 × 10^7^ cells/mL cancer cells grown in logarithmic phase were harvested and resuspended in FBS-free 1640 medium. Then, 100 μL of the cell suspension was subcutaneously injected into the right flank of each mouse. When the tumour volume was reached about 600–800 mm^3^, it was removed, minced into small pieces of equal volume (2 × 2 × 2 mm^3^), and transplanted subcutaneously into the nude mice. When the tumour volume was reached about 200–400 mm^3^, the xenograft tumour-bearing nude mice were randomly allocated to five groups (vehicle-treated, 1D228-2mgd-treated, 1D228-4mgd-treated 1D228-4mgd-treated and Tepotinib-treated groups, with 6 or 8 mice (The number of mice were determined according to previous experience on xenograft tumor models) per group. Each group was dosed by intragastric administration (ig) for 7–14 days. Tumour volume and body weights were recorded every day after drug treatment. At the end of the observation period, the animals were euthanized by cervical dislocation and the tumour bulks were peeled off conformed to the Guide for the Care and Use of Laboratory Animals as published by the US National Institutes of Health (NIH Publication No. 85-23, revised 1996) and approved by the Institutional Ethics Review Board of Binzhou Medical University. There was no blinding was done during the animal experiment. No animals were excluded from the analysis.

#### Acute toxicity test

Female and male Kunming mice (36 mice each, 18 ~ 25 g) were purchased and housed at the SPF (Beijing) Biotechnology Co. in pathogen-free condition, maintained at constant room temperature and fed a standard rodent chow and water. The mice were randomly divided into experimental group and control group. The animals were fasted overnight before drug administration, and the drug was administered by tail vein injection at a constant volume of 0.4 ml/20 g (concentration of the administered drug: 17.5, 55, 175, 550, 750 mg/kg) in two doses within 24 h, with an interval of 6 h. Acute toxicity of the animals was observed for 7 days after the administration of the drug. The heart, liver, lung and kidney of mice in the 750 mg/kg dose group were taken and fixed for HE staining.

#### Statistical analysis

All experiments were repeated three times independently unless specifically indicated. The data was analyzed by GraphPad Prism 7 software. The data was shown as mean ± SD and one-way ANOVA Tukey ‘s multiple comparison test was used to determine the levels of significance between comparison samples. *p* > 0.05 was not significant (ns). *p < 0.05; **p < 0.01; ***p < 0.001.

### Supplementary information

SupplementaI Information

Original Data File

aj-checklist

## Data Availability

Raw sequence data from this study were deposited in the NCBI Short Read Archive (SRA; http://www.ncbi.nlm.nih.gov/sra) under the accession number of PRJNA1002561.
