# Supplementary material for: A novel c-Met/TRK inhibitor 1D228 efficiently inhibits tumor growth by targeting angiogenesis and tumor cell proliferation
Source: Cell Death Dis. 2023 Nov 9;14(11):728. doi: 10.1038/s41419-023-06246-5 (PMC10636171; doi:10.1038/s41419-023-06246-5)
Supplement: Supplementary file 1 — SupplementaI Information [file 41419_2023_6246_MOESM1_ESM.docx]

A novel c-Met/TRK inhibitor 1D228 efficiently inhibits tumor growth by targeting angiogenesis and tumor cell proliferation.

Baijiao An^1,3^, Wenyan Nie^1^ , Jinhui Hu^4^, Yangyang Fan^1^, Haoran Nie^1^, Mengxuan Wang^1^, Yaxuan Zhao^1^, Han Yao^2^, Yuanyuan Ren^2^, Chuanchuan Zhang^1^, Mengna Wei^2^, Wei Li^2^, Jiadai Liu^2^, Chunhua Yang ^1^, Yin Zhang ^1,^^3*^, Xingshu Li^2*^, and Geng Tian^1,3*^.

1 *School of Pharmacy,* *Binzhou Medical University, Yantai, Shandong, 264003, PR China*

*2 School of Pharmaceutical Sciences, Sun Yat-Sen University, Guangzhou 510006, PR China*

*3 Shandong Technology Innovation Center of Molecular Targeting and Intelligent Diagnosis and Treatment, Yantai, Shandong, 264003, PR China*

*4 School of Biotechnology and Health Sciences,* *Wuyi University, Jiangmen, 529020, PR China*

**Supplementary Contents**

**Content**

1. **Supplemental Figures and legends**
2. **Supplemental Tables**
3. **Original Western blot**

**
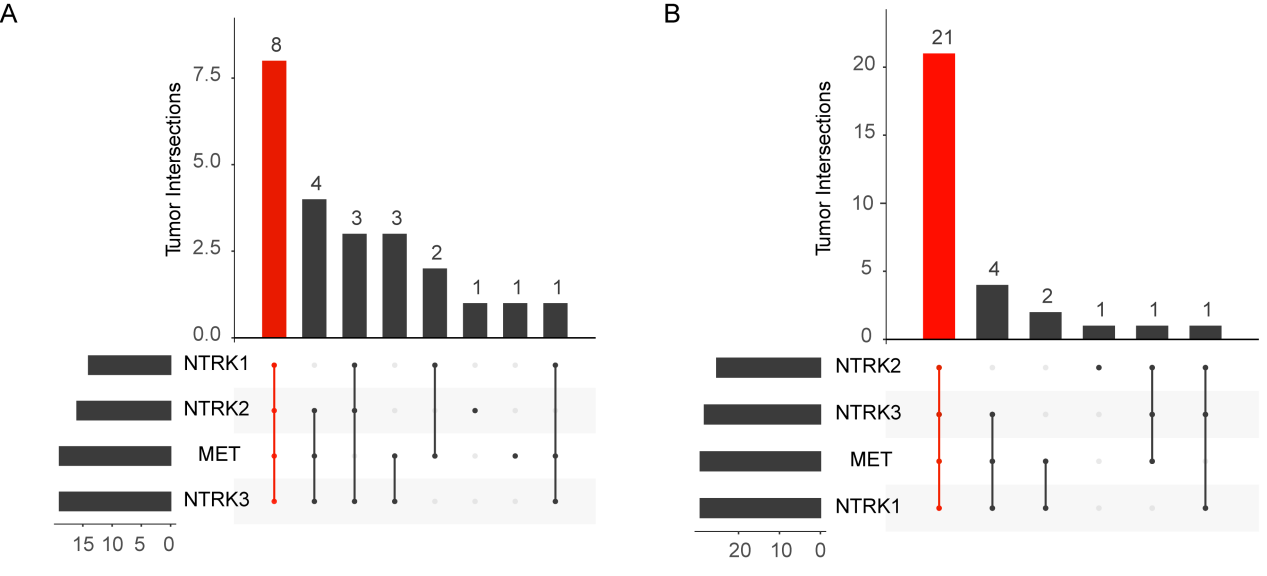
**

Fig.S1 Upset maps show the simultaneous and significantly abnormal intersection of MET, NTRK1, NTRK2, and NTRK3 expression in multiple cancers; (A) 8 cancer species in the TCGA database showed significant abnormal expression of both NTRK and c-Met；(B) 21 cancer species in TCGA and GTEx database showed significant aberrant expression of both of them.


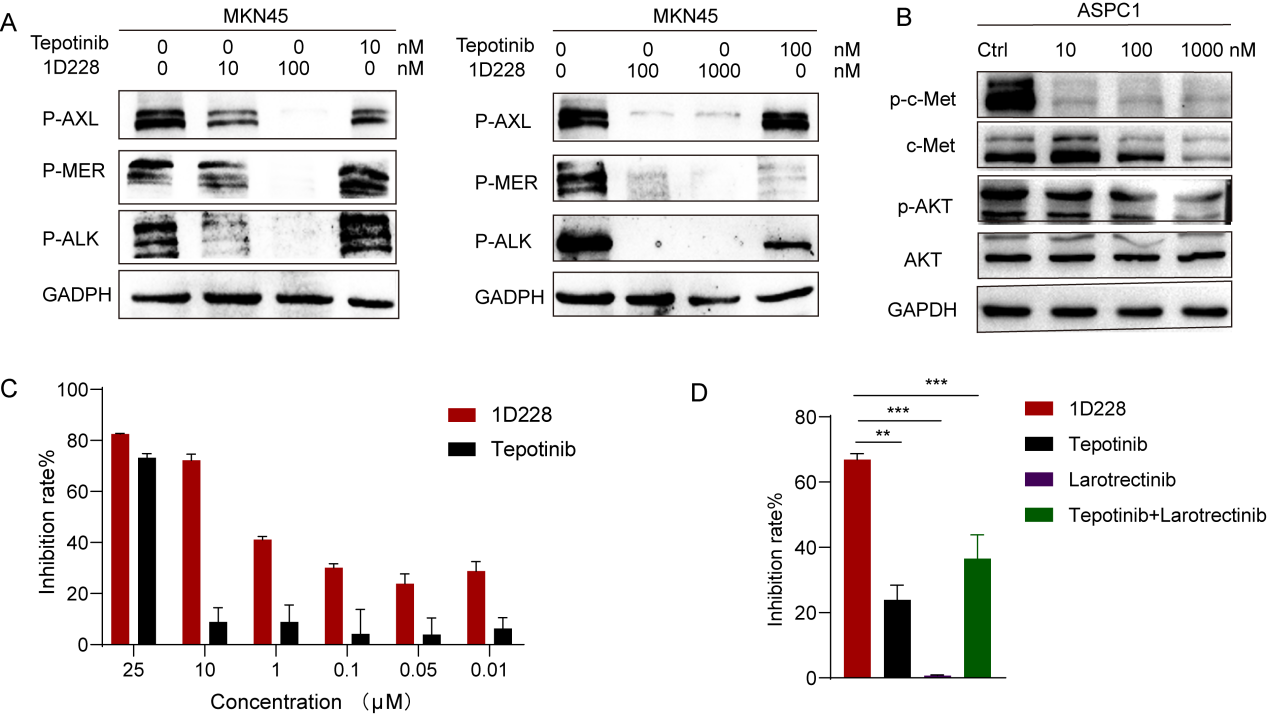


Fig.S2 Effects of 1D228 on the signaling pathway and anti-proliferative activity of MKN45, ASPC1 and HS746T cells. (A) Expression of AXL, p-AXL, ALK, p-ALK, MER and p-MER proteins in MKN45 cells；(B) Effect of 1D228 on the expression of c-Met, AKT and their corresponding phosphorylated proteins in ASPC1 cells；(C) Activity of 1D228 on HS746T; (D) CCK8 assay for anti-proliferative activity of 1D228, Tepotinib, Larotrectinib and combinations against MKN45 (Data are represented as mean ± SD, n=3. Unpaired t-test was used. ***: P≤0.001, **: P≤0.01).


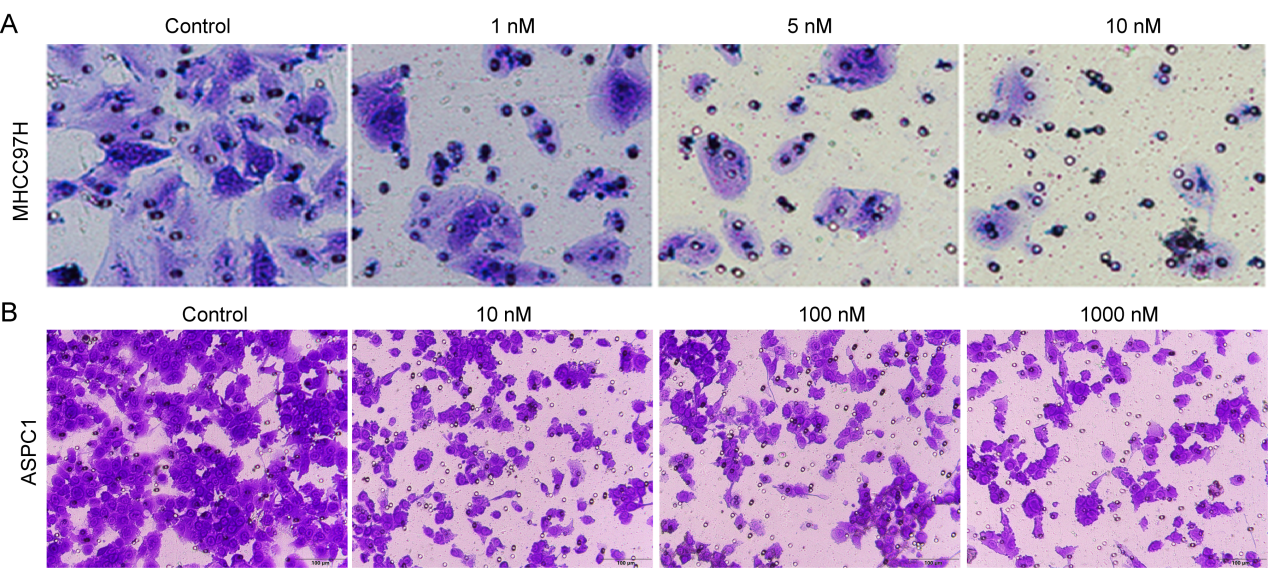


Fig.S3 1D228 inhibited the migration of MHCC97H and ASPC1 cells. (A) 1D228 inhibited the migration of MHCC97H cells at concentrations of 1, 5 and 10 nM; (B) 1D228 inhibited the migration of ASPC1 cells at concentrations of 10, 100 and 100 nM.


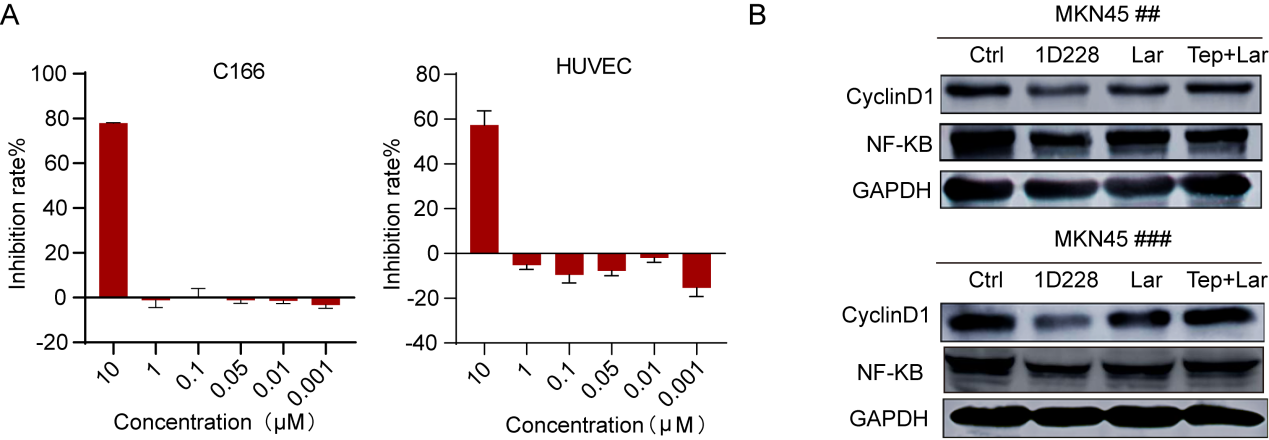


Fig.S4 (A)Antiproliferative activity of the compound 1D228 against C166 cells and HUVEC cells; (B) Protein level of CyclinD1 and NF-KB in MKN45 tumor tissues.


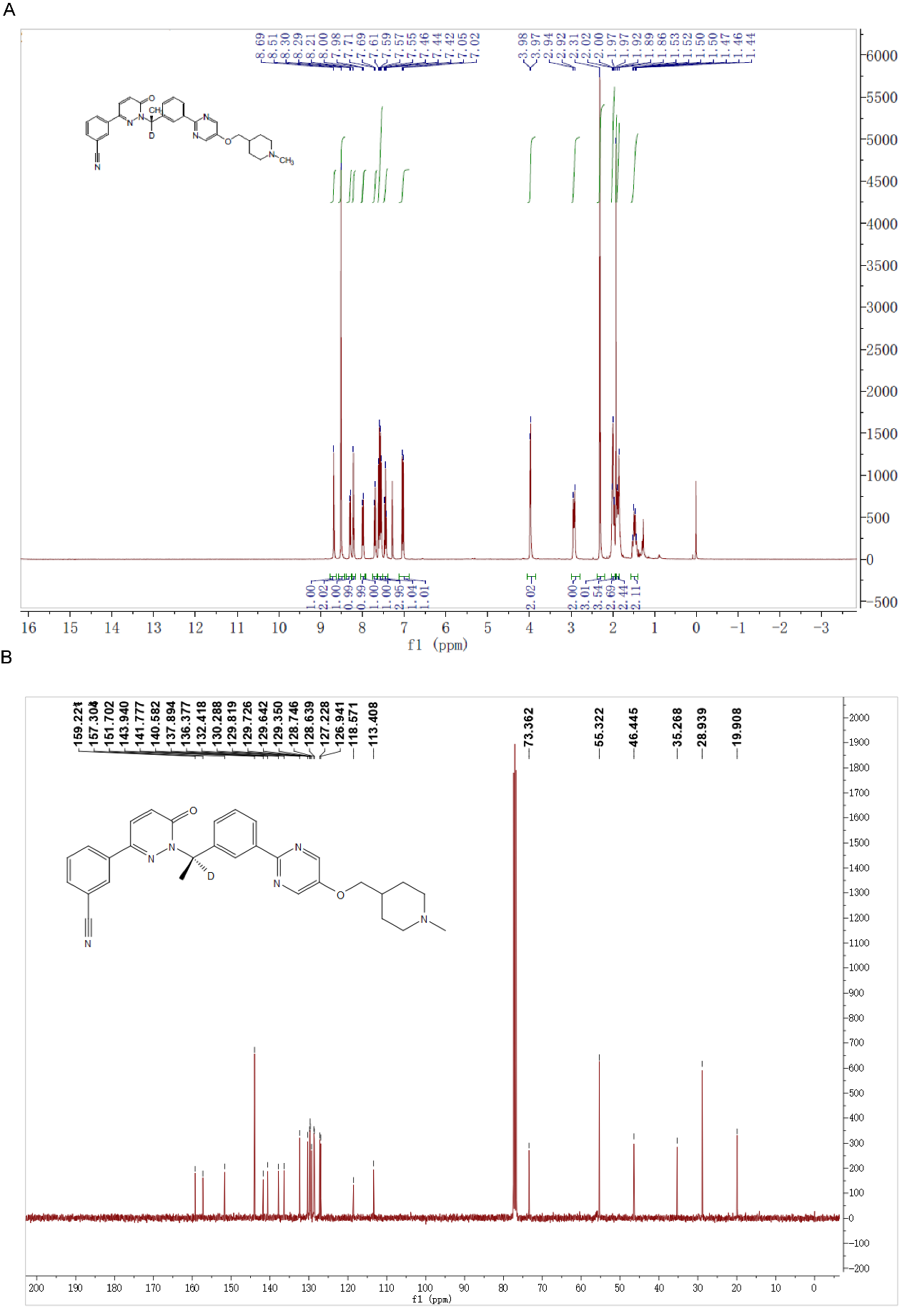


Fig.S5 ^1^H NMR (A) and ^13^C NMR (B) of 1D228.


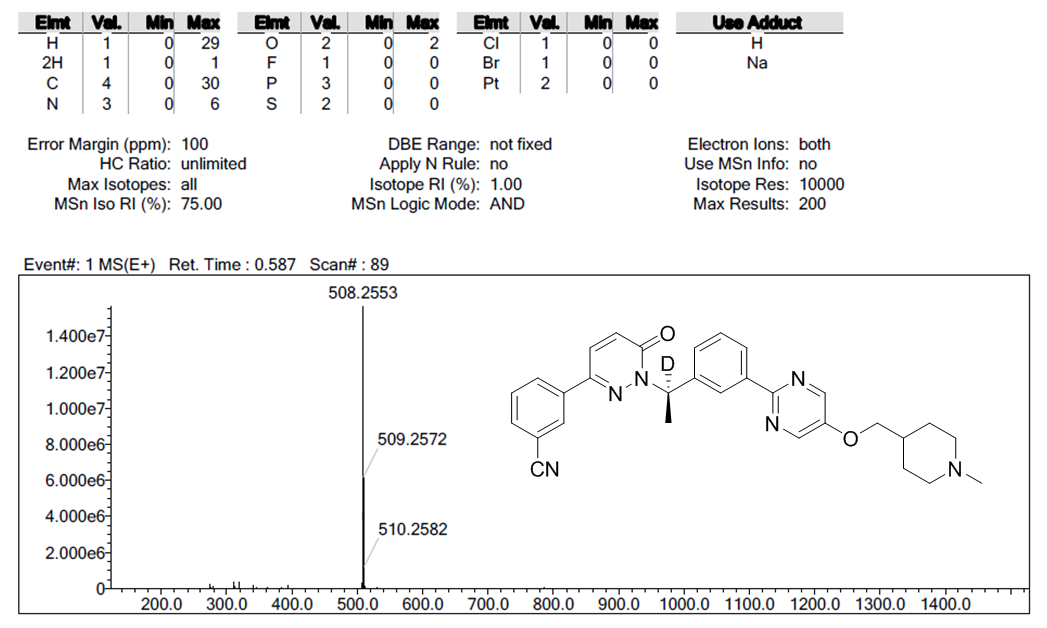


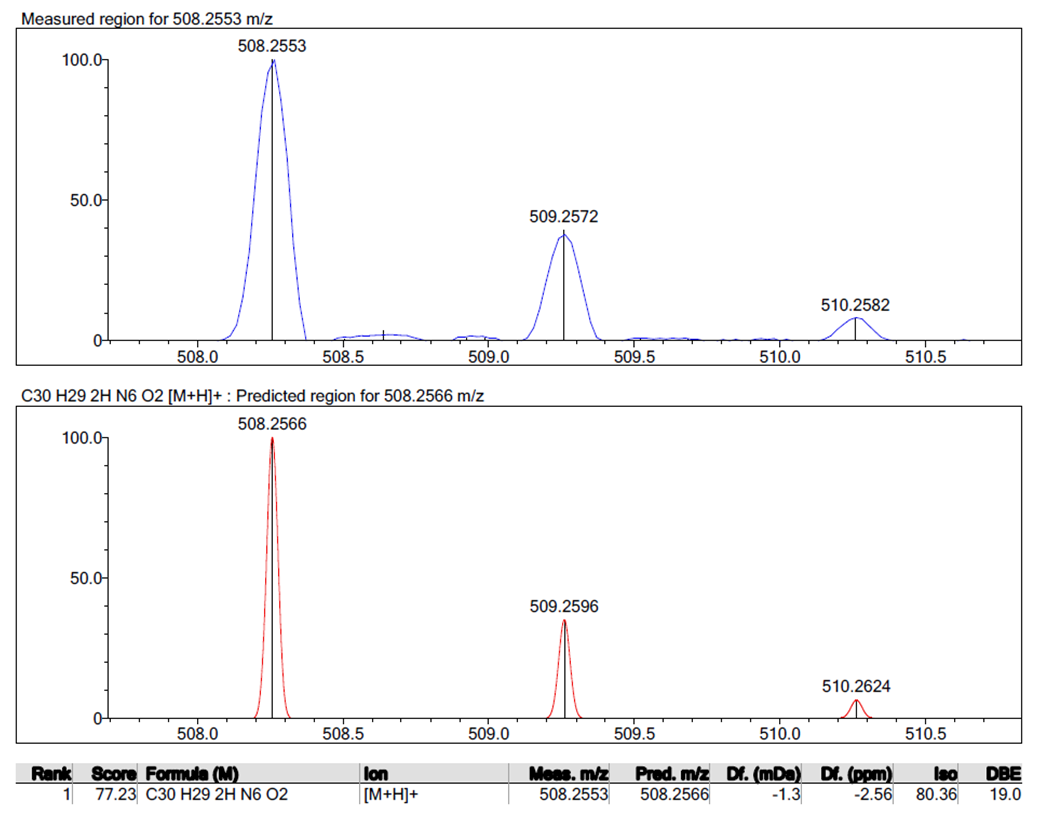


Fig.S6 HRMS (ESI) Spectrum of 1D228.

**Supplemental tables**

Table S1 The TCGA database reveals abnormal expression of MET, and NTRKs simultaneously in 8 cancer species

| MET | NTRK1 | NTRK2 | NTRK3 | ..count.. | values |
| --- | --- | --- | --- | --- | --- |
| TRUE | TRUE | TRUE | TRUE | 8 | BLCA、GBM、KICH、KIRC、KIRP、LUAD、PRAD、THCA |
| FALSE | TRUE | TRUE | TRUE | 3 | LUSC、UCEC、 |
| TRUE | FALSE | TRUE | TRUE | 4 | BRCA、COAD、LIHC、READ |
| FALSE | FALSE | TRUE | TRUE | 0 |  |
| TRUE | TRUE | FALSE | TRUE | 1 | HNSC |
| FALSE | TRUE | FALSE | TRUE | 0 |  |
| TRUE | FALSE | FALSE | TRUE | 3 | CESC、ESCA、STAD |
| FALSE | FALSE | FALSE | TRUE | 0 |  |
| TRUE | TRUE | TRUE | FALSE | 0 |  |
| FALSE | TRUE | TRUE | FALSE | 0 |  |
| TRUE | FALSE | TRUE | FALSE | 0 |  |
| FALSE | FALSE | TRUE | FALSE | 1 | THYM |
| TRUE | TRUE | FALSE | FALSE | 2 | CHOL、PCPG |
| FALSE | TRUE | FALSE | FALSE | 0 |  |
| TRUE | FALSE | FALSE | FALSE | 1 | PAAD |

Table S2 Significant aberrant expression of METs, and NTRKs simultaneously in 21 cancer species in the TCGA and GTEx database

| MET | NTRK1 | NTRK2 | NTRK3 | ..count.. | values |
| --- | --- | --- | --- | --- | --- |
| TRUE | TRUE | TRUE | TRUE | 21 | ACC、BLCA、BRCA、CESC、COAD、GBM、KICH、KIRC、KIRP、LGG、LIHC、LUAD、LUSC、OV、PAAD、PRAD、READ、SKCM、TGCT、THCA、UCEC |
| FALSE | TRUE | TRUE | TRUE | 1 |  |
| TRUE | FALSE | TRUE | TRUE | 1 | DLBC |
| FALSE | FALSE | TRUE | TRUE | 0 |  |
| TRUE | TRUE | FALSE | TRUE | 4 | ESCA、HNSC、STAD、UCS |
| FALSE | TRUE | FALSE | TRUE | 0 |  |
| TRUE | FALSE | FALSE | TRUE | 0 |  |
| FALSE | FALSE | FALSE | TRUE | 0 |  |
| TRUE | TRUE | TRUE | FALSE | 0 |  |
| FALSE | TRUE | TRUE | FALSE | 0 |  |
| TRUE | FALSE | TRUE | FALSE | 0 |  |
| FALSE | FALSE | TRUE | FALSE | 1 | THYM |
| TRUE | TRUE | FALSE | FALSE | 2 | CHOL、PCPG |
| FALSE | TRUE | FALSE | FALSE | 0 |  |
| TRUE | FALSE | FALSE | FALSE | 0 |  |

Table S3 Active compound 1D228 selectivity profile against 77 kinases

| No. | Kinase | 1D228(%inhibition)@500nM | SD |
| --- | --- | --- | --- |
| 1 | TRKC | 98.97490899 | 0.516729044 |
| 2 | MET | 98.41023795 | 0.123434959 |
| 3 | TRKB | 97.14737997 | 0.242132851 |
| 4 | TRKA | 93.58061047 | 0.333267742 |
| 5 | ALK | 89.49144648 | 2.570688057 |
| 6 | AXL | 89.15455865 | 0.586372084 |
| 7 | MER | 84.12182119 | 2.583330992 |
| 8 | LYNa | 27.34466781 | 11.56474128 |
| 9 | INSR | 18.55089981 | 0.862983345 |
| 10 | FLT3 | 17.85846483 | 1.58932497 |
| 11 | RET | 12.2397088 | 3.465533248 |
| 12 | FRK | 11.30581833 | 7.741350166 |
| 13 | YES | 11.30093532 | 1.954908286 |
| 14 | EGFR T790M L858R | 11.10777471 | 0.474070641 |
| 15 | FYN [b] | 10.89557436 | 3.792222137 |
| 16 | MUSK | 9.134475789 | 3.315180022 |
| 17 | HER2 | 8.776664097 | 1.986036229 |
| 18 | EPHA8 | 8.524810723 | 0.384258597 |
| 19 | TEC | 8.286494281 | 0.318370202 |
| 20 | PDGFRα | 8.205647675 | 6.968805766 |
| 21 | JAK1 | 7.194830582 | 0.714171706 |
| 22 | ROS1 | 7.047430651 | 1.950648344 |
| 23 | ABL1 | 6.373033883 | 18.31837362 |
| 24 | FLT1 | 6.00455485 | 8.897028373 |
| 25 | EPHB3 | 5.67500008 | 0.926656354 |
| 26 | KDR | 5.618924349 | 1.265862957 |
| 27 | LYNb | 5.264063305 | 0.70167176 |
| 28 | IGF1R | 5.259258184 | 0.37436658 |
| 29 | LCK | 5.206929863 | 7.789832508 |
| 30 | BMX | 5.121428705 | 0.896501642 |
| 31 | EPHB4 | 5.077790615 | 0.572159301 |
| 32 | TYK2 | 4.509326659 | 4.568177769 |
| 33 | ABL2 | 4.452347197 | 8.340013503 |
| 34 | TYRO3 | 4.202290908 | 1.590761847 |
| 35 | IRR | 3.882700344 | 0.984574313 |
| 36 | EPHA5 | 3.739816649 | 1.555792545 |
| 37 | CSF1R | 3.21373716 | 1.038892456 |
| 38 | BRK | 3.18869635 | 1.47802798 |
| 39 | FGFR3 | 3.051062478 | 0.15681926 |
| 40 | SRC | 2.90585978 | 2.585173437 |
| 41 | SRM | 2.655628234 | 1.204384972 |
| 42 | SYK | 2.468116698 | 2.438437437 |
| 43 | Her4 | 2.211068735 | 0.594009271 |
| 44 | ACK | 1.959804148 | 1.022349265 |
| 45 | JAK2 | 1.822938633 | 2.400345295 |
| 46 | FGFR1 | 1.720749484 | 4.351100522 |
| 47 | ITK | 1.600503327 | 8.697459449 |
| 48 | RON | 1.3838053 | 1.444041213 |
| 49 | TXK | 1.291750327 | 4.877836911 |
| 50 | EGFR | 1.173340299 | 0.419443343 |
| 51 | PYK2 | 1.141612741 | 0.28602106 |
| 52 | FES | 1.046088687 | 0.892904376 |
| 53 | FGFR2 | 1.036313628 | 1.947712304 |
| 54 | JAK3 | 0.193346546 | 0.781065561 |
| 55 | FGFR4 | -0.369902136 | 3.19016627 |
| 56 | ZAP70 | -0.65998976 | 2.292060251 |
| 57 | EPHA2 | -0.797754207 | 4.338051332 |
| 58 | EPHA7 | -1.356870217 | 0.204220471 |
| 59 | EPHA1 | -1.533013103 | 1.271117261 |
| 60 | EPHA6 | -1.78052958 | 2.853206283 |
| 61 | TIE2 | -2.642591574 | 2.662917088 |
| 62 | FLT4 | -3.030927591 | 4.024762545 |
| 63 | CSK | -3.072334066 | 4.867674947 |
| 64 | EPHB2 | -3.544633411 | 1.722118302 |
| 65 | BTK | -3.812926293 | 0.465931217 |
| 66 | PDGFRβ | -3.951967635 | 4.942501836 |
| 67 | DDR2 | -3.977464913 | 5.064065849 |
| 68 | HCK | -4.058094123 | 9.038667158 |
| 69 | KIT | -4.496350796 | 4.960618953 |
| 70 | EPHB1 | -6.478447133 | 7.523719008 |
| 71 | FYN [a] | -7.775129935 | 1.531977428 |
| 72 | FAK | -9.578633085 | 7.56830502 |
| 73 | EPHA3 | -11.81050837 | 6.590522602 |
| 74 | DDR1 | -11.81297028 | 7.985033977 |
| 75 | FER | -13.29509443 | 3.82347252 |
| 76 | EPHA4 | -14.66809515 | 2.775240101 |
| 77 | FGR | -20.70240676 | 5.279764343 |

Table S4 The 80 pairs samples in  tissue microarrays for immunohistochemistry analysis

| NO. | Gender | Age | Tissue type | Pathological classification | Grade | Expression gene | |
| --- | --- | --- | --- | --- | --- | --- | --- |
| 1 | Male | 40 | Tumor | gastric adenocarcinoma | Ⅲ | TRKlow | METhigh |
| 2 | Male | 45 | Tumor | gastric adenocarcinoma | Ⅱ-Ⅲ | TRKlow | METhigh |
| 3 | Male | 76 | Tumor | gastric adenocarcinoma | Ⅱ | TRKhigh | METhigh |
| 4 | Female | 56 | Tumor | gastric adenocarcinoma | Ⅲ | TRKlow | METhigh |
| 5 | Male | 75 | Tumor | gastric adenocarcinoma | Ⅲ | TR Klow | METhigh |
| 6 | Male | 69 | Tumor | gastric adenocarcinoma | Ⅲ | TRKhigh | METhigh |
| 7 | Male | 68 | Tumor | gastric adenocarcinoma | Ⅱ-Ⅲ | TRKlow | METhigh |
| 8 | Male | 46 | Tumor | gastric adenocarcinoma | Ⅱ | TRKlow | METhigh |
| 9 | Male | 68 | Tumor | gastric adenocarcinoma | Ⅲ | TRKlow | METhigh |
| 10 | Male | 56 | Tumor | gastric adenocarcinoma | Ⅱ-Ⅲ | TRKlow | METlow |
| 11 | Female | 72 | Tumor | gastric adenocarcinoma | Ⅱ-Ⅲ | TRKhigh | METhigh |
| 12 | Male | 71 | Tumor | gastric adenocarcinoma | Ⅱ | TRKlow | METlow |
| 13 | Male | 63 | Tumor | gastric adenocarcinoma | Ⅱ-Ⅲ | TRKlow | METhigh |
| 14 | Male | 63 | Tumor | gastric adenocarcinoma | Ⅲ | TRKlow | METlow |
| 15 | Female | 65 | Tumor | gastric adenocarcinoma | Ⅱ-Ⅲ | TRKlow | METhigh |
| 16 | Male | 55 | Tumor | gastric adenocarcinoma | Ⅱ | TRKlow | METhigh |
| 17 | Male | 79 | Tumor | gastric adenocarcinoma | Ⅲ | TRKlow | METhigh |
| 18 | Male | 77 | Tumor | gastric adenocarcinoma | Ⅱ | TRKlow | METhigh |
| 19 | Male | 52 | Tumor | gastric adenocarcinoma | Ⅱ-Ⅲ | TRKlow | METlow |
| 20 | Male | 65 | Tumor | gastric adenocarcinoma | Ⅲ | TRKlow | METlow |
| 21 | Female | 71 | Tumor | gastric adenocarcinoma | Ⅱ | TRKlow | METhigh |
| 22 | Male | 70 | Tumor | gastric adenocarcinoma | Ⅱ | TRKlow | METhigh |
| 23 | Male | 62 | Tumor | gastric adenocarcinoma | Ⅲ | TRKlow | METhigh |
| 24 | Female | 54 | Tumor | gastric adenocarcinoma | Ⅲ | TRKlow | METhigh |
| 25 | Male | 65 | Tumor | gastric adenocarcinoma | Ⅱ | TRKhigh | METhigh |
| 26 | Male | 61 | Tumor | gastric adenocarcinoma | Ⅲ | TRKlow | METlow |
| 27 | Male | 77 | Tumor | gastric adenocarcinoma | Ⅲ | TRKlow | METlow |
| 28 | Male | 75 | Tumor | gastric adenocarcinoma | Ⅱ-Ⅲ | TRKlow | METlow |
| 29 | Male | 66 | Tumor | gastric adenocarcinoma | Ⅱ | TRKlow | METhigh |
| 30 | Male | 60 | Tumor | gastric adenocarcinoma | Ⅲ | TRKlow | METhigh |
| 31 | Male | 72 | Tumor | gastric adenocarcinoma | Ⅱ | TRKlow | METhigh |
| 32 | Female | 83 | Tumor | gastric adenocarcinoma | Ⅱ | TRKlow | METhigh |
| 33 | Male | 69 | Tumor | gastric adenocarcinoma | Ⅱ | TRKlow | METlow |
| 34 | Male | 60 | Tumor | gastric adenocarcinoma | Ⅲ | TRKlow | METlow |
| 35 | Female | 80 | Tumor | gastric adenocarcinoma | Ⅲ | TRKhigh | METlow |
| 36 | Male | 53 | Tumor | gastric adenocarcinoma | Ⅱ | TRKlow | METlow |
| 37 | Male | 53 | Tumor | gastric adenocarcinoma | Ⅱ-Ⅲ | TRKlow | METlow |
| 38 | Male | 52 | Tumor | gastric adenocarcinoma | Ⅲ | TRKlow | METlow |
| 39 | Male | 58 | Tumor | gastric adenocarcinoma | Ⅱ | TRKlow | METhigh |
| 40 | Male | 65 | Tumor | gastric adenocarcinoma | Ⅱ | TRKlow | METhigh |
| 41 | Female | 73 | Tumor | gastric adenocarcinoma | Ⅱ | TRKlow | METhigh |
| 42 | Male | 67 | Tumor | gastric adenocarcinoma | Ⅱ | TRKhigh | METhigh |
| 43 | Male | 77 | Tumor | gastric adenocarcinoma | Ⅱ-Ⅲ | TRKlow | METhigh |
| 44 | Male | 48 | Tumor | gastric adenocarcinoma | Ⅱ-Ⅲ | TRKlow | METhigh |
| 45 | Female | 62 | Tumor | gastric adenocarcinoma | Ⅲ | TRKlow | METlow |
| 46 | Female | 34 | Tumor | gastric adenocarcinoma | Ⅲ | TRKlow | METlow |
| 47 | Male | 85 | Tumor | gastric adenocarcinoma | Ⅱ-Ⅲ | TRKlow | METhigh |
| 48 | Male | 59 | Tumor | gastric adenocarcinoma | Ⅲ | TRKlow | METlow |
| 49 | Male | 68 | Tumor | gastric adenocarcinoma | Ⅱ-Ⅲ | TRKlow | METhigh |
| 50 | Male | 50 | Tumor | gastric adenocarcinoma | Ⅱ-Ⅲ | TRKlow | METlow |
| 51 | Male | 51 | Tumor | gastric adenocarcinoma | Ⅱ | TRKlow | METhigh |
| 52 | Male | 72 | Tumor | gastric adenocarcinoma | Ⅲ | TRKlow | METlow |
| 53 | Male | 73 | Tumor | gastric adenocarcinoma | Ⅱ | TRKlow | METhigh |
| 54 | Male | 70 | Tumor | gastric adenocarcinoma | Ⅱ | TRKlow | METhigh |
| 55 | Female | 53 | Tumor | gastric adenocarcinoma | Ⅱ-Ⅲ | TRKlow | METlow |
| 56 | Male | 69 | Tumor | gastric adenocarcinoma | Ⅱ-Ⅲ | TRKlow | METlow |
| 57 | Male | 67 | Tumor | gastric adenocarcinoma | Ⅱ | TRKlow | METhigh |
| 58 | Male | 65 | Tumor | gastric adenocarcinoma | Ⅲ | TRKlow | METlow |
| 59 | Male | 55 | Tumor | gastric adenocarcinoma | Ⅲ | TRKlow | METlow |
| 60 | Female | 85 | Tumor | gastric adenocarcinoma | Ⅱ | TRKlow | METhigh |
| 61 | Male | 30 | Tumor | gastric adenocarcinoma | Ⅲ | TRKlow | METhigh |
| 62 | Male | 73 | Tumor | gastric adenocarcinoma | Ⅱ-Ⅲ | TRKlow | METhigh |
| 63 | Female | 64 | Tumor | gastric adenocarcinoma | Ⅱ-Ⅲ | TRKlow | METlow |
| 64 | Female | 67 | Tumor | gastric adenocarcinoma | Ⅱ-Ⅲ | TRKlow | METlow |
| 65 | Male | 64 | Tumor | gastric adenocarcinoma | Ⅲ | TRKlow | METlow |
| 66 | Male | 56 | Tumor | gastric adenocarcinoma | Ⅱ-Ⅲ | TRKhigh | METhigh |
| 67 | Male | 63 | Tumor | gastric adenocarcinoma | Ⅱ-Ⅲ | TRKlow | METhigh |
| 68 | Female | 54 | Tumor | gastric adenocarcinoma | Ⅱ-Ⅲ | TRKlow | METlow |
| 69 | Male | 60 | Tumor | gastric adenocarcinoma | Ⅲ | TRKlow | METlow |
| 70 | Male | 60 | Tumor | gastric adenocarcinoma | Ⅱ-Ⅲ | TRKlow | METhigh |
| 71 | Male | 60 | Tumor | gastric adenocarcinoma | Ⅱ | TRKhigh | METhigh |
| 72 | Male | 86 | Tumor | gastric adenocarcinoma | Ⅱ | TRKlow | METhigh |
| 73 | Male | 53 | Tumor | gastric adenocarcinoma | Ⅲ | TRKlow | METhigh |
| 74 | Male | 64 | Tumor | gastric adenocarcinoma | Ⅱ-Ⅲ | TRKlow | METlow |
| 75 | Male | 53 | Tumor | gastric adenocarcinoma | Ⅱ | TRKlow | METhigh |
| 76 | Male | 72 | Tumor | gastric adenocarcinoma | Ⅲ | TRKlow | METhigh |
| 77 | Male | 51 | Tumor | gastric adenocarcinoma | Ⅱ | TRKlow | METlow |
| 78 | Female | 43 | Tumor | gastric adenocarcinoma | Ⅲ | TRKlow | METlow |
| 79 | Female | 75 | Tumor | gastric adenocarcinoma | Ⅲ | TRKlow | METlow |
| 80 | Male | 56 | Tumor | gastric adenocarcinoma | Ⅱ-Ⅲ | TRKlow | METlow |
