# Supplementary figures and images for: A novel c-Met/TRK inhibitor 1D228 efficiently inhibits tumor growth by targeting angiogenesis and tumor cell proliferation
Source: Cell Death Dis. 2023 Nov 9;14(11):728. doi: 10.1038/s41419-023-06246-5 (PMC10636171; doi:10.1038/s41419-023-06246-5)

**Original Western blot**

**
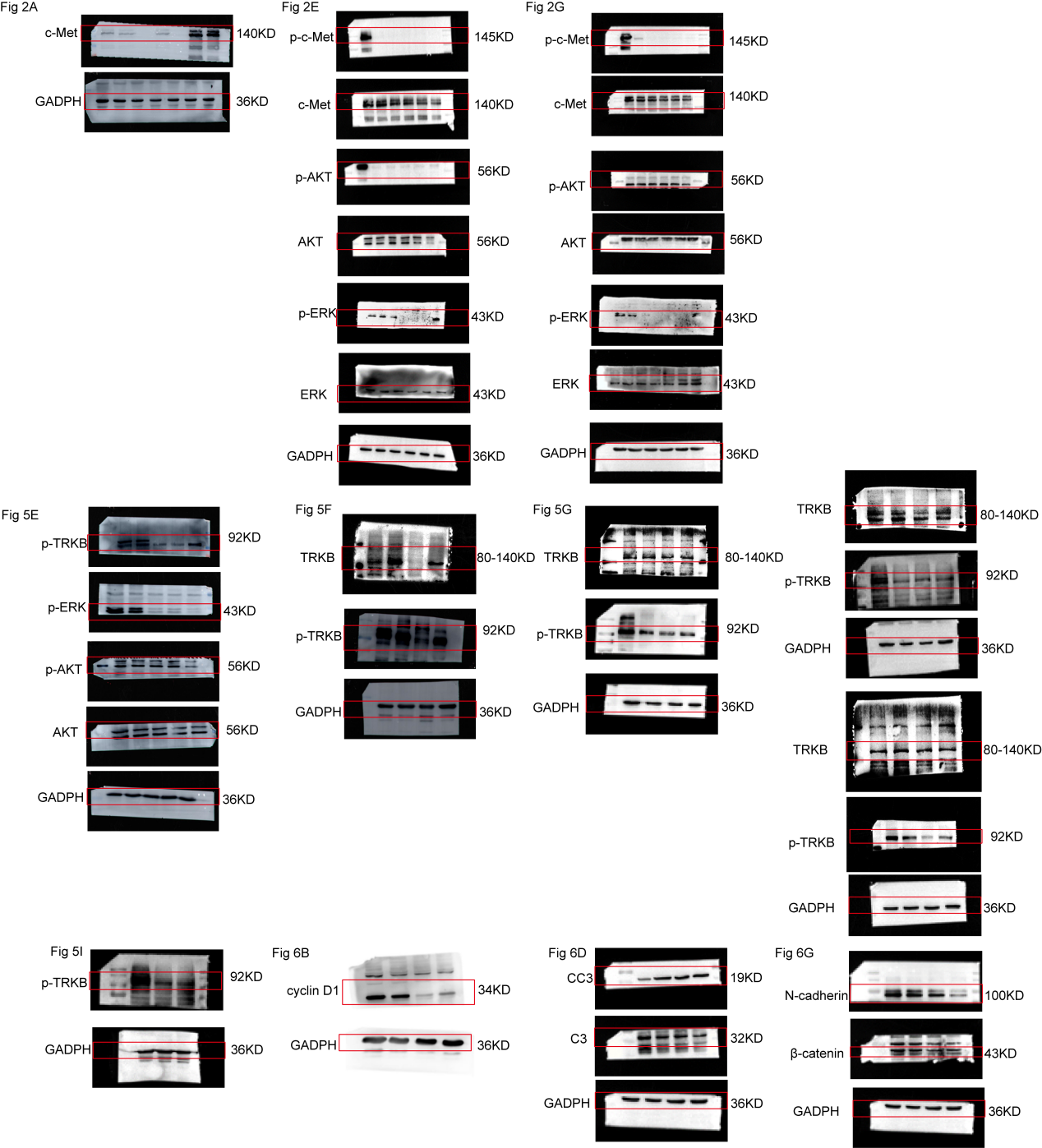
**


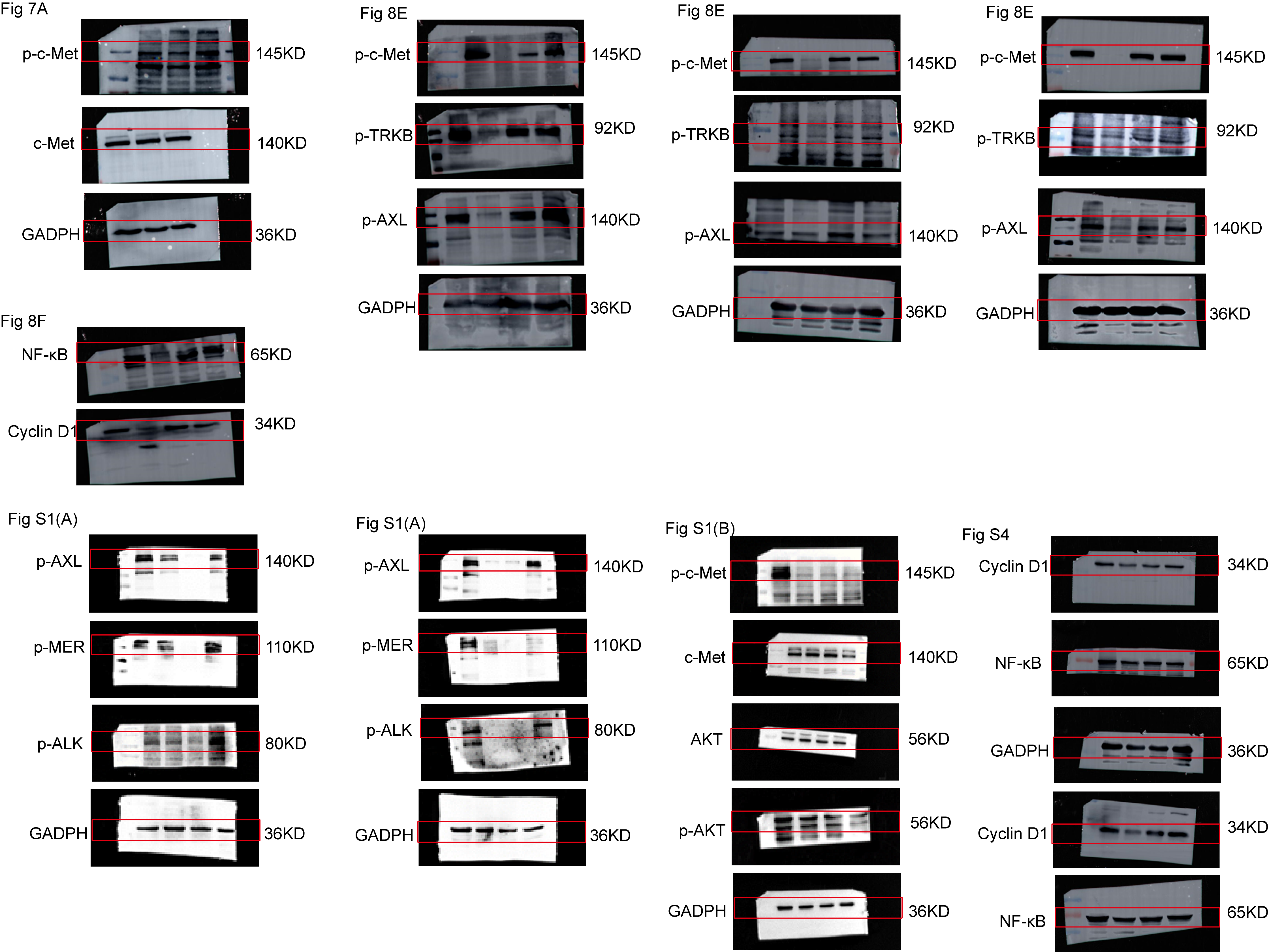

Supplement: Supplementary file 2 — Original Data File [file 41419_2023_6246_MOESM2_ESM.docx]
